# Comprehensive genomic profiling reveals prognostic signatures and insights into the molecular landscape of colorectal cancer

**DOI:** 10.3389/fonc.2023.1285508

**Published:** 2023-11-13

**Authors:** Jinwei Yang, Sihui Zhao, Junyan Su, Siyao Liu, Zaozao Wu, Wei Ma, Ming Tang, Jingcui Wu, Erdong Mao, Li Han, Mengyuan Liu, Jiali Zhang, Lei Cao, Jingyi Shao, Yun Shang

**Affiliations:** ^1^ Second Department of General Surgery, The First People’s Hospital of Yunnan Province, The Affiliated Hospital of Kunming University of Science and Technology, Kunming, China; ^2^ Institute of Neuroscience, Kunming Medical University, Kunming, China; ^3^ Department of Scientific Research Projects, Beijing ChosenMed Clinical Laboratory Co. Ltd., Beijing, China; ^4^ Department of Pathology, The First People’s Hospital of Yunnan Province, The Affiliated Hospital of Kunming University of Science and Technology, Kunming, China; ^5^ Department of Reproductive Medicine, The First People’s Hospital of Yunnan Province, The Affiliated Hospital of Kunming University of Science and Technology, Kunming, China

**Keywords:** colorectal cancer, next generation sequencing, signature, prognosis, protein validation

## Abstract

**Background:**

Colorectal cancer (CRC) is a prevalent malignancy with diverse molecular characteristics. The NGS-based approach enhances our comprehension of genomic landscape of CRC and may guide future advancements in precision oncology for CRC patients.

**Method:**

In this research, we conducted an analysis using Next-Generation Sequencing (NGS) on samples collected from 111 individuals who had been diagnosed with CRC. We identified somatic and germline mutations and structural variants across the tumor genomes through comprehensive genomic profiling. Furthermore, we investigated the landscape of driver mutations and their potential clinical implications.

**Results:**

Our findings underscore the intricate heterogeneity of genetic alterations within CRC. Notably, *BRAF*, *ARID2*, *KMT2C*, and *GNAQ* were associated with CRC prognosis. Patients harboring *BRAF*, *ARID2*, or *KMT2C* mutations exhibited shorter progression-free survival (PFS), whereas those with *BRAF*, *ARID2*, or *GNAQ* mutations experienced worse overall survival (OS). We unveiled 80 co-occurring and three mutually exclusive significant gene pairs, enriched primarily in pathways such as TP53, HIPPO, RTK/RAS, NOTCH, WNT, TGF-Beta, MYC, and PI3K. Notably, co-mutations of *BRAF*/*ALK*, *BRAF*/*NOTCH2*, *BRAF*/*CREBBP*, and *BRAF*/*FAT1* correlated with worse PFS. Furthermore, germline *AR* mutations were identified in 37 (33.33%) CRC patients, and carriers of these variants displayed diminished PFS and OS. Decreased AR protein expression was observed in cases with *AR* germline mutations. A four-gene mutation signature was established, incorporating the aforementioned prognostic genes, which emerged as an independent prognostic determinant in CRC via univariate and multivariate Cox regression analyses. Noteworthy BRAF and ARID2 protein expression decreases detected in patients with their respective mutations.

**Conclusion:**

The integration of our analyses furnishes crucial insights into CRC’s molecular characteristics, drug responsiveness, and the construction of a four-gene mutation signature for predicting CRC prognosis.

## 1 Introduction

Colorectal cancer (CRC) is the third most common cancer worldwide (colon: 1,148,515; rectum: 732,210) (1) and the second leading cause of cancer-related deaths (colon: 935,000; rectum: 339,022) (2) in 2020. There were about 555,000 new cases of colorectal cancer in China in 2020, accounting for 12.2% of the number of new cancers in that year, and nearly 286,000 deaths, accounting for 9.5% of the number of cancer deaths in China that year (3). The five-year survival rates for colon and rectal cancers were 64.4% and 66.6%, respectively (4). Approximately 20% of CRC patients typically present with metastasis at diagnosis, and over 50% of CRC patients experience metastasis during their disease (5). The majority of metastatic CRC (mCRC) patients are rarely cured. However, patients with isolated liver and/or lung metastases, limited local recurrence, or restricted peritoneal spread can be cured through a multidisciplinary approach, including surgery. Treatment for other patients with mCRC is palliative systemic chemotherapy, with a median overall survival (OS) of approximately 30 months (6).

In recent years, key research areas in CRC have focused on the application of immunotherapy and the study of related biomarkers, exploration of the relationship between the gut microbiome and cancer, improvement of early screening and diagnostic methods, development of personalized treatment strategies, research into new drugs and treatment modalities, and the identification and validation of biomarkers (7–11). These research directions hold the promise of improving the treatment outcomes and prognosis for colorectal cancer patients.The current biomarkers employed by clinicians to forecast the prognosis and therapeutic responsiveness of CRC patients, such as carcinoembryonic antigen (CEA) and carbohydrate antigen 19-9 (CA19-9), exhibit relatively limited sensitivity and specificity (12). Novel types of biomarkers are arising. Molecular-based biomarkers, such as genetic markers, epigenetic markers, and their signatures, enable physicians to more precisely stratify patients for personalized treatment (13).

With the continuous development of genetic testing technology, several next-generation sequencing (NGS) studies have revealed the genomics profile of primary and metastatic CRC (14–16). Customized therapeutic approaches have been proposed for particular patients, guided by their inherent molecular characteristics, including oncogenic mutations, tumor mutation burden (TMB), and microsatellite instability (MSI) status. Approximately 35% of CRC tissues carry an active mutation in exon 2 (codons 12/13) of *KRAS*, which does not benefit from EGFR monoclonal antibody (6). Approximately 10% to 15% of mCRC patients harbor BRAF mutations, with V600E being the predominant type. *BRAF* V600E exhibits a high degree of mutual exclusivity with KRAS and NRAS mutations (17). It leads to the continuous activation of the MAPK signaling pathway, resulting in heightened clinical aggressiveness and resistance towards monoclonal antibody therapies targeting *EGFR*. Moreover, *BRAF* V600E is closely linked to unfavorable survival outcomes in mCRC patients (18).

Approximately 15% of CRCs harbored deficient mismatch repair (dMMR) or high MSI (MSI-H). CRCs exhibiting dMMR or MSI-H are marked by elevated TMB, resulting in an abundant of neoantigens that provoke a vigorous immune reaction within the tumor microenvironment, involving tumor-infiltrating lymphocytes (19). The findings from the KEYNOTE 177 clinical trial demonstrated the potential advantages of immune checkpoint inhibitors (ICIs) for patients with MSI-H and dMMR CRC (20). The FDA approved two PD-1 inhibitors, nivolumab, and pembrolizumab, for treating chemotherapy of refractory CRC with dMMR/MSI-H.

In this study, we conducted a prospective clinical sequencing analysis involving 111 patients at the First People’s Hospital of Yunnan Province. Utilizing a comprehensive NGS panel comprising 599 genes, we extensively analyzed clinical information, treatment outcomes, and genomics profile. Our study reveals that *BRAF, ARID2*, *KMT2C*, and *GNAQ* are prognostic biomarkers for CRC.

## 2 Materials and methods

### 2.1 Samples source and ethical data

In this study, 111 patients diagnosed with CRC were collected at First People’s Hospital of Yunnan Province (FPHYP) between July 10, 2019, and October 24, 2022. Tissues and paired peripheral blood samples were collected for NGS testing. For every patient, detailed clinicopathological attributes, such as age, gender, tumor location, tumor size, tumor differentiation, tumor stage, first-line therapy, and follow-up duration, were accessible and recorded (**Additional file 2: Table S1**). Tumor staging was conducted following the 8th edition of the AJCC staging system for CRC. All samples were approved and authorized by the First People’s Hospital of Yunnan Province Ethics Committee, and all participants provided informed consent.

### 2.2 DNA extraction and sequencing

Genomic DNA extraction from FFPE tissues was performed in accordance with the manufacturer’s guidelines using the Maxwell RSC FFPE plus DNA kit (Promega, Cat no. AS1720). For DNA extraction from peripheral blood lymphocytes, the Blood gDNA purification kit (Concert, Cat: RC1001) was utilized. Subsequently, 100ng of genomic DNA was subjected to shearing, targeting fragment sizes of 200 bp, using the Covaris E210 system (Covaris, Inc.). We performed next-generation sequencing of tumor and gDNA-matched germline DNA for library preparation using KAPA HyperPrep Kit (Roche,07962312001) and Agilent SureSelect XT kit (Agilent, G9702C). Following library preparation, library quantification was conducted using the Qubit 3.0 Fluorometer (Life Technologies, Inc.), and assessment of quality and fragment size was performed using the Agilent 2100 Bioanalyzer (Agilent Technologies, Inc.). Subsequently, the samples underwent paired-end sequencing on an Illumina Nova Seq 6000 platform (Illumina Inc., USA), with reads spanning 150 base pairs. The raw Illumina sequence data underwent demultiplexing and subsequent conversion into fastq files. Post-adaptor removal and trimming of low-quality sequences, the obtained qualified reads were employed for various analyses, including somatic variants, germline variants, microsatellite instability analysis, mutational signature analysis, and fusion genes analysis, all as outlined below.

### 2.3 Data processing

The raw sequencing data underwent alignment against the reference human genome (UCSC hg19) using the Burrows-Wheeler Aligner. Subsequently, duplicate reads were eliminated, and local realignment procedures were executed. The Genome Analysis Toolkit (GATK) was utilized for calling single nucleotide variations (SNVs), insertions, and deletions (indels). Following this, germline alterations were removed by comparing the matched blood samples, resulting in the identification of somatic alterations. To annotate the variants, the ANNOVAR software tool was employed. Copy number analysis was conducted using CNVkit. The genomic alteration, clinicopathological characteristics, and survival data of Metastatic Colorectal Cancer (MSK, Cancer Cell 2018) and Colorectal Cancer (MSK, Gastroenterology 2020) from the Memorial Sloan Kettering Cancer Center (MSKCC) database were downloaded from cBioPortal (http://www.cbioportal.org).

### 2.4 Somatic variants

Somatic variants displaying allele frequencies (AF) exceeding 0.5% were derived from each tumor genomic DNA samples by excluding germline variants. These identified somatic variants were subsequently annotated using the Catalog of Somatic Mutations in Cancer (COSMIC) database. The functional categorization of each somatic mutation adhered to the interpretation and reporting standards and guidelines set forth by the Association for Molecular Pathology, American Society of Clinical Oncology, and College of American Pathologists (ASCO/CAP). The TMB was assessed by quantifying the number of nonsynonymous somatic mutations within the targeted gene sequencing region. The TMB value of less than 10 mut/mb is categorized as ‘TMB-L,’ while a TMB value of 10 mut/mb or greater is categorized as ‘TMB-H’. Additionally, a TMB value of 100 mut/mb or higher is classified as hypermutation.

### 2.5 Germline variants

Germline variants were identified in gDNA from buffy coat fraction aliquots if their allele frequency (AF) exceeded 25%. Variants with a frequency of over 1% in the Exome Aggregation Consortium database (ExAC) were excluded from consideration. The integrated details of germline mutations filtered workflow is summarized as follows:

Genomic variants are initially called using Genome Analysis Toolkit (GATK v4.2.2.0) HaplotypeCaller (https://gatk.broadinstitute.org/hc/en-us/articles/360037225632-HaplotypeCaller). Subsequently, variants are subjected to VariantFiltration, utilizing GATK’s recommended parameters. Only variants marked as “PASS” in the VCF file are retained, ensuring the inclusion of high-confidence variants. The annotated VCF file is further processed using three software tools: Annovar (v20191024), SnpEff (v4.3), and VEP (release-96). Annotation provides insights into the functional impact of variants, including whether they are non-synonymous mutations or occur within coding regions. To narrow down the variants to those of clinical relevance, a set of stringent filtering criteria is applied. This includes the exclusion of synonymous mutations and deletions/insertions (delins) longer than 50 base pairs. Variants with a sequence coverage of less than 50 base pairs are filtered out. Variants with a population frequency greater than1% in databases such as the 1000 Genomes Project, Exome Aggregation Consortium (ExAC), and esp6500siv2 are excluded. Additionally, variants classified as benign or likely benign in ClinVar and variants with a frequency lower than 25% are filtered out.

The assessment of germline variants conformed to the established standards and guidelines defined by the American College of Medical Genetics and Genomics and the Association for Molecular Pathology (ACMG/AMP). Moreover, the interpretation underwent independent review by two genetic consultants to verify its precision and dependability.

### 2.6 Microsatellite instability analysis

To determine each patient’s MSI status, we utilized MSIsensor (21). In this study, the software was utilized to analyze the length distributions of microsatellites at specific locations in both the paired tumor and matched-normal BAM files. The observed distributions in both samples were subjected to statistical comparison. The analysis involved identifying the total number of sites with sufficient data (requiring at least 20 spanning reads in normal and tumor samples) and then calculating the number of somatic sites. The MSI score was subsequently calculated, representing the percentage of somatic sites. As the algorithm recommended, samples with an MSI score equal to or exceeding 20% were classified as ‘MSI-H’.

### 2.7 Mutational signature analysis

The FPHYP cohort, consisting of 111 tumors, employed the Non-negative matrix factorization approach described by Alexandrov et al. (22), to infer mutation signatures. This approach utilized 96 mutational contexts derived from SNVs caused by six base substitutions (C > A, C > G, C > T, T > A, T > C, and T > G) within 16 possible combinations of neighboring bases for each substitution. These mutational contexts were employed as input data for the estimation of their respective contributions to the observed mutations. Additionally, a comparative analysis was conducted between the newly identified mutation signatures and 30 established cancer signatures sourced from the COSMIC database (23). This comparison was conducted to determine the exposure contributions of the inferred mutation signatures to the observed mutations.

### 2.8 Fusion gene testing

Fusion gene identification was carried out through RNA-based methodologies in this study. RNA sequencing facilitates the quantification of mRNA levels and the detection and investigation of gene fusions, alternative gene splicing events, transcript modifications, and disease-associated single nucleotide polymorphisms across the entire transcriptome, encompassing noncoding RNA regions (24). To detect fusion genes, specialized software tools of STAR-Fusion were employed (25). Functional annotation and pertinent details regarding the fusion genes were ascertained by referencing Ensembl and RefSeq gene databases. The linkage between fusion genes and diseases was also established by consulting disease databases such as Online Mendelian Inheritance in Man (OMIM, https://www.omim.org/) and Catalogue of Somatic Mutations in Cancer (COSMIC, https://cancer.sanger.ac.uk/cosmic).

### 2.9 Immunohistochemical staining evaluation

The expression levels of PD-L1 were evaluated using immunohistochemistry (IHC) to determine the patient’s suitability for immunotherapy. Formalin-fixed, paraffin-embedded (FFPE) tissues were cut into 4 µm thick slices. The tissue slices were then subjected to incubation with an anti-PD-L1 antibody (DAKO, 22C3) at a dilution of 1:100, following the manufacturer’s recommended protocols for immunostaining. Blinded to the identity of the samples, two pathologists conducted independent evaluations and quantifications of staining results. This involved determining both the Tumor Proportion Score (TPS) and Combined Positive Score (CPS). The TPS is calculated as the ratio of stained tumor cells to the total tumor cell count within the sample, while the CPS entails dividing the sum of PD-L1 positive cells, encompassing both tumor and immune cells, by the total tumor cell count and then multiplying by 100 to express it as a percentage. A TPS ≥1% or CPS ≥ 1 was considered positive, indicating the presence of PD-L1 expression.

In addition, IHC for BRAF, ARID2, and AR protein expression was performed in FFPE tumor sections. Staining was performed with BRAF antibody (Bioss, Beijing, China), ARID2 antibody (Boster, CA, USA), and AR (Bioss, Beijing, China) to demonstrate protein expression, respectively. Slides were incubated with the BRAF antibody (diluted 1:100), ARID2 antibody (diluted 1:200), and AR antibody (diluted 1:100) at four °C overnight. The protein expression was detected with the DAB Detection kit (Zsbio). Following chromogenic detection, all slides were counterstained with Hematoxylin II and Bluing Reagent (Ventana) for 5 minutes each, and coverslips were applied. All immunostained slides were evaluated independently by two pathologists. All stained slides were scanned using a Holographic scanner (3DHISTECH). Then, the images of each sample slide were analyzed using Image Pro Plus 6.0 (Media Cybernetics, Inc). Cumulative optical density (IOD) represents the cumulative sum of fluorescence intensity within an image. The IOD/Area value was calculated for each slide of the tumor region. Concordance between immunohistochemically analyzed BRAF, ARID2, and AR protein expression and mutation status and clinicopathological variables was analyzed using t-test or Chi-Square, as appropriate.

### 2.10 Statistical analysis

All statistical analyses were performed in this study using R software (version 4.2.2, Institute for Statistics and Mathematics, Vienna, Austria). Statistical comparisons between two groups were performed using Fisher’s test, t-test, or Chi-Square test as appropriate. Survival differences were evaluated using the Kaplan-Meier approach, and the significance was determined using the log-rank test. Univariate and multivariate analyses were carried out employing the Cox proportional hazard regression model. All statistical tests were two-sided, and a p-value of less than 0.05 was considered to indicate statistical significance.

## 3 Results

### 3.1 Genomics characteristics of Chinese CRC patients

**Figure 1:**
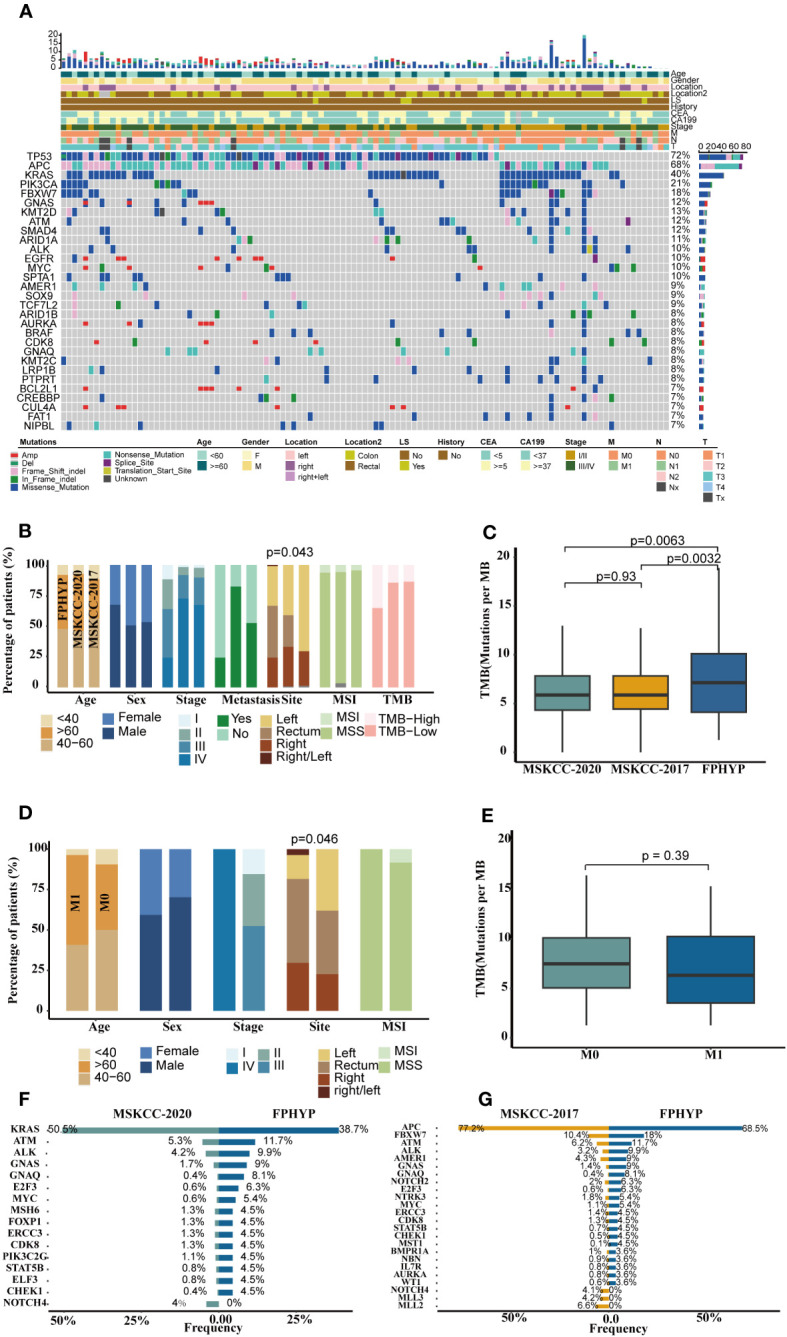
Genomics profile of FPHYP cohort. **(A)** Mutation landscape and associated clinicopathological characteristics of 111 CRC patients. **(B)** Comparison of clinical features among FPHYP, MSKCC-2020, and MSKCC-2017 cohorts. **(C)** Comparison of TMB among three cohorts. **(D)** Comparison of clinical characteristics between mCRC and non-mCRC groups in FPHYP cohort. **(E)** Comparison of TMB between non-mCRC and mCRC in FPHYP cohort. **(F, G)** Differentially mutated genes between FPHYP and MSKCC-2020 **(F)** or MSKCC-2017 cohorts **(G)**. TMB, tumor mutation burden; mCRC, metastatic CRC; non-mCRC, non-metastatic CRC.

A cohort of 111 CRC cases from the FPHYP was enrolled, and the clinicopathologic features are summarized in **Table 1**. The tumor and paired peripheral blood samples were performed with ChosenOne^®^ Pan-cancer genetic testing protocol, which utilizes a panel of 599 genes (**Additional file 2: Table S2**). The most frequently cancer-associated genes in this cohort were *TP53* (72%), *APC* (68%), *KRAS* (40%), *PIK3CA* (21%), *FBXW7* (18%), *GNAS* (12%), *KMT2D* (13%), *ATM* (12%), *SMAD4* (12%), and *ARID1A* (11%) (**Figure 1A**). The clinicopathological attributes set the FPHYP cohort apart from both the MSKCC-2020 and MSKCC-2017 cohorts (**Figure 1B**). Among the three cohorts, the FPHYP cohort had the highest proportion of males (67.57% vs. 50.74% vs. 53.33%; p = 0.102), fewest stage IV patients (24.32% vs. 72.61% vs. 67.43%; p = 0.333), the lowest ratio of mCRC patients (24.32% vs. 82.38% vs. 52.63%; p = 0.156), the highest proportion of rectal cancer cases (42.34% vs. 25.90% vs. NA; p = 0.044), and most TMB-high patients (35.14% vs. 14.44% vs. 13.60%, p = 0.277; **Figure 1B**). The FPHYP cohort cases harbored the highest tumor mutation burden (TMB) among the three cohorts (**Figure 1C**). Compared with non-metastatic CRC (non-mCRC) in the FPHYP cohort, the mCRC group had more elder patients (55.56% vs. 40.74% vs. 3.70%; p = 0.275), more females (p = 0.121), higher proportion rectum cases (p = 0.046), and with all MSS patients (**Figure 1D**). The TMB in the mCRC group was decreased compared with those in the non-mCRC group of FPHYP cohort (p = 0.36, **Figure 1E**). *ATM*, *ALK*, *GNAS*, *GNAQ*, *E2F3*, *MYC*, *FOXP1*, *ERCC3*, *CDK8*, *PIK3C2G*, *STAT5B*, *ELF3*, and *CHEK1* genes were significantly highly enriched in FPHYP cohort compared to MSKCC-2020 (**Figure 1F**).While *FBXW7*, *ATM*, *ALK*, *GNAS*, *GNAQ*, *NOTCH2*, *E2F3*, *NTRK3*, *ERCC3*, *CDK8*, *STAT5B*, *CHEK1, and MST1* genes were significantly highly enriched in FPHYP cohort compared to MSKCC-2017 (**Figure 1G**).

**Table 1:** Clinicopathological characteristics of 111 CRC patients in the FPHYP cohort.

Total patients	N = 111	(%)
Age		
median	59 (17–87)	/
Gender		
Male	75	67.57
Female	36	32.43
AJCC TNM stage		
I	13	11.71
II	27	24.32
III	44	39.64
IV	27	24.32
Primary Site		
Left colon	36	32.43
Right colon	27	24.32
Rectum	47	42.34
Others	1	0.90
Histology		
adenocarcinoma	68	61.26
adenocarcinoma with mucinous component	7	6.31
mucinous adenocarcinoma	6	5.41
adenocarcinoma with signet ring cell	1	0.90
Intramucosal carcinoma	1	0.90
Others or Unknown	28	25.23
Histological Differentiation		
moderately	57	51.35
moderately-highly	2	1.80
moderately-poorly	13	11.71
poorly	13	11.71
poorly-moderately	3	2.70
NA and others	23	20.72
MSI status		
MSS	104	93.69
MSI	7	6.31
Unknown	0	0.00
TMB status		
TMB-Low	72	64.86
TMB-High	39	35.14
Metastasis status		
Yes	27	24.32
No	84	75.68
First-line Therapy		
Chemotherapy	65	58.56
Chemotherapy + bevacizumab/cetuximab	14	12.61
Unknown	32	28.83

### 3.2 Prognostic significance of *BRAF*, *ARID2*, *KMT2C*, and *GNAQ*

**Figure 2:**
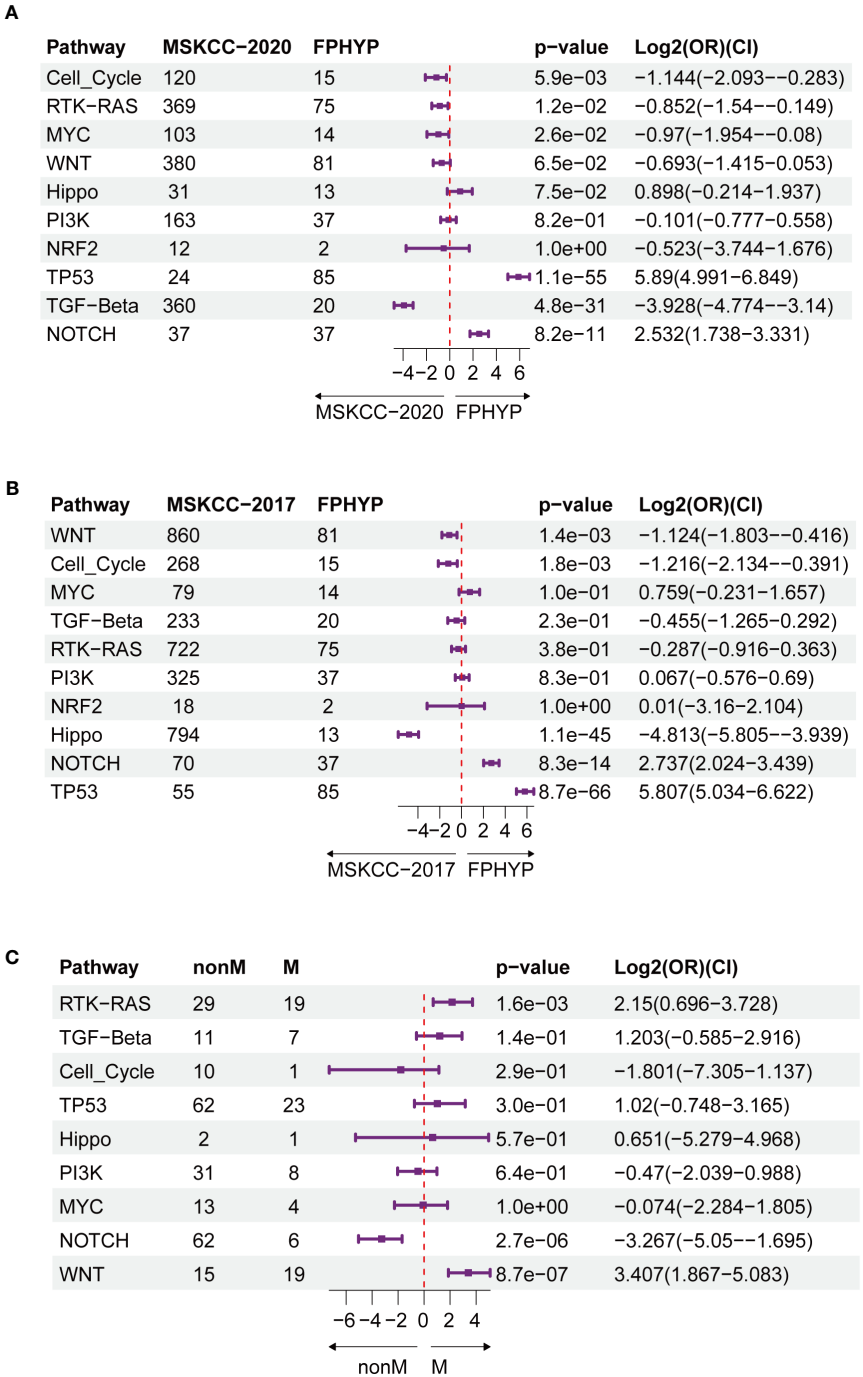
Oncogenic signaling pathways analysis between FPHYP and MSKCC cohorts. **(A)** Contrasting the mutation statuses of oncogenic signaling pathways between the FPHYP and MSKCC-2020 cohorts. **(B)** Analyzing the mutation statuses of oncogenic signaling pathways in both the FPHYP and MSKCC-2017 cohorts. **(C)** Comparison of the mutation status of oncogenic signaling pathways between non-mCRCs and mCRCs in the FPHYP cohort. Assessing the mutation statuses of oncogenic signaling pathways in non-mCRC and mCRC groups within the FPHYP cohort. mCRC, metastatic CRC; non-mCRC, non-metastatic CRC.

**Figure 3:**
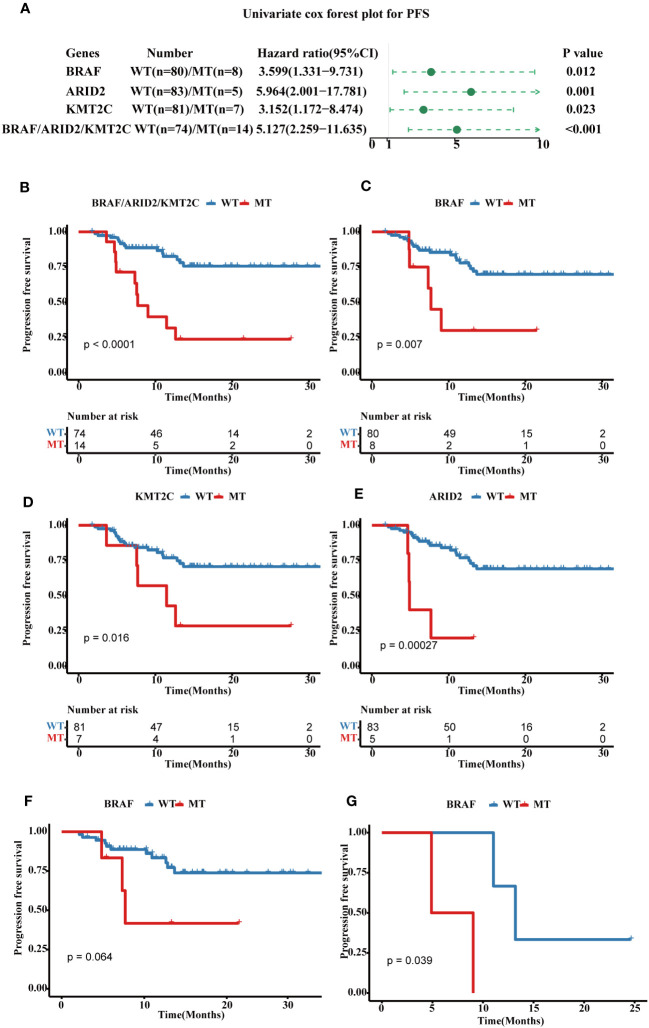
Prognostic associated somatic mutated genes in FPHYP CRC cohort. **(A)** Univariable analyses of PFS concerning somatic gene mutations in FPHYP CRC tumors. **(B)** Kaplan-Meier curves for PFS between three genes(*BRAF*, *ARID2*, and *KMT2C*) combined MT and WT groups. **(C–E)** Kaplan-Meier curves for PFS based on *BRAF* **(C)**, *ARID2* **(D)**, and *KMT2C* **(E)** mutation status. **(F, G)** Kaplan-Meier plots of PFS for CRC patients undergoing exclusive first-line chemotherapy **(F)** and chemotherapy combined with bevacizumab **(G)**, stratified by *BRAF* mutation status. PFS, progression-free survival; MT, mutation type; WT, wiled type.

A higher genomic alterations incidence of the TP53 (p < 0.0001), NOCTH (p < 0.0001), and Hippo (p = 0.075) pathways were discovered in FPHYP than in the MSKCC-2020 cohort (**Figure 2A**). A higher genomic alterations incidence of the NOCTH (p < 0.0001) and TP53 (p < 0.0001) pathways were discovered in FPHYP than in MSKCC-2017 cohorts (**Figure 2B**). Compared with non-mCRC tumors, the alterations incidence of RTK-RAS (p = 0.0016) and WNT (p < 0.0001) pathways were higher in the mCRC group in the FPHYP cohort (**Figure 2C**). The molecular profiles of CRC in relation to of PFS were explored. Univariate analyses of PFS showed that tumors with *BRAF* mutations (hazard ratio, 3.599; 95% CI, 1.331–9.731; p = 0.012), *ARID2* mutations (hazard ratio, 5.964; 95% CI, 2.001–17.781; p = 0.001), and *KMT2C* mutations (hazard ratio, 3.152; 95% CI, 1.172–8.474; p = 0.023) were at high risk of metastasis or recurrence (**Figure 3A**). The patients of the three genes combined MT group (harbored mutations of *BRAF, ARID2*, and/or *KMT2C*) also showed shorter PFS than the three genes combined WT group(without any mutations of *BRAF, ARID2*, and *KMT2C*) (**Figure 3B**). The survival analyses showed that patients with mutation type (MT) in *BRAF*, *ARID2*, and *KMT2C* group had shorter progression-free survival (PFS) than the respectively wild type (WT) patients (p-value: 0.007, 0.00027, and 0.016, respectively, **Figures 3C–E**), which was consistent with previous reports (26). Among cases of receiving only chemotherapy CRC, the *BRAF*-mutant group exhibited a decreased progression-free survival (PFS) compared to the wild-type subgroup (median PFS: 6.97 months vs. 13.20 months; p = 0.064) (**Figure 3F**). Significantly, in the case of the two patients harboring a *BRAF* mutation, the inclusion of bevacizumab in the initial chemotherapy regimen, either FOLFOX (fluoropyrimidine-based agents plus leucovorin and oxaliplatin) or CAPAOX (capecitabine plus oxaliplatin), did not result in improved outcomes (p = 0.039, **Figure 3G**).

**Figure 4:**
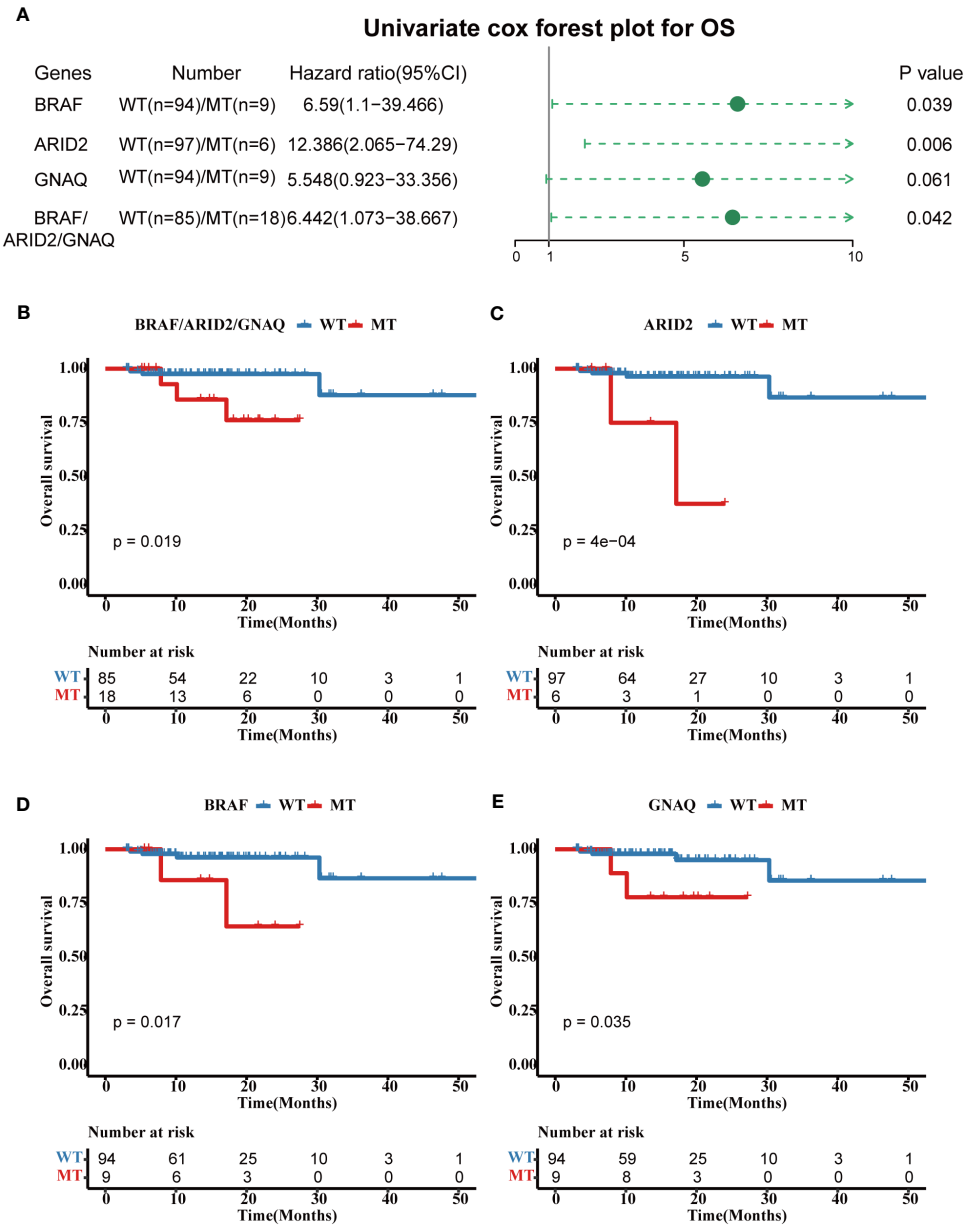
Prognostic associated somatic mutated genes in FPHYP CRC cohort. **(A)** Univariable analyses of OS concerning somatic gene mutations in FPHYP CRC tumors. **(B)** Kaplan-Meier curves for OS between three genes(*BRAF*, *ARID2*, and *KMT2C*) combined MT and WT groups. **(C–E)** Kaplan-Meier curves for PFS based on *BRAF* **(C)**, *ARID2* **(D)**, and *KMT2C* **(E)** mutation status. OS, overall survival; MT, mutation type; WT, wiled type.

Univariate analyses of overall survival (OS) revealed that tumors with *BRAF* mutations (hazard ratio, 6.56; 95% CI, 1.1–39.466; p = 0.039), *ARID2* mutations (hazard ratio, 12.386; 95% CI, 2.065–74.29; p = 0.006), and *GNAQ* mutations (hazard ratio, 5.548; 95% CI, 0.923–33.356; p = 0.061) were associated with poor prognosis (**Figure 4A**). For OS, the cases in three genes combined mutation group (harbored mutations of *BRAF, ARID2*, and/or *GANQ*) also showed shorter OS than those in the three genes combined WT group (without any mutations of *BRAF, ARID2*, and *GANQ*) (**Figure 4B**). The survival analyses showed that patients with MT in *BRAF*, *ARID2*, and *GANQ* group had shorter OS than the respectively WT patients (p-value: 0.017, 4e-04, and 0.035, respectively, **Figures 4C–E**), which was consistent with previous reports (27). The analysis of the association between clinical features and alterations in these four genes revealed a significant correlation between *KMT2C* mutation status and gender. Additionally, a statistically significant relationship was observed between *ARID2* mutation status and MSI status (**Additional file 2: Table S3**).

In addition, *KRAS*, *CDK8*, *SMAD4*, *BRAF*, *GANQ, E2F3, MYC, NRAS, CHEK1*, and *KMT5A* were identified as diver genes (**Additional file 1: Figure S1A**). In the FPHYP cohort, ten somatic variations of *BRAF* were identified in nine CRCs patients, including V600E (n = 6), G758R (n = 1), P403fs (n = 1), and L584I co-occurrence with R389C (n = 1) (**Additional file 1: Figure S1C**). 5.4% (6 of 111) of patients harbored nine *ARID2* mutations (**Additional file 1: Figure S1D**), 8.1% (9 of 111) of patients harbored fourteen *KMT2C* mutations (**Figure S1E**), 8.1% (9 of 111) of patients harbored nine *GNAQ* mutations (**Additional file 1: Figure S1F**).

In the FPHYP cohort, three denovo signatures were identified using SomaticSignatures R package. The cosine similarity between these three signatures and 30 Catalogue of Somatic Mutations in Cancer (COSMIC) signatures are shown in **Figure S2A** (**Additional file 1**). Signature 1 exhibited the highest similarity (cosine similarity of 0.859) with COSMIC_3 and is primarily linked to defects in DNA double-strand break repair by homologous recombination (HR) (**Additional file 1: Figure S2B**). Signature 2 showed the strongest resemblance (cosine similarity of 0.93) to COSMIC_6 and is mainly associated with defects in DNA mismatch repair (**Additional file 1: Figure S2B**). Signature 3 displayed the closest match (cosine similarity of 0.989) with COSMIC_10 and is primarily linked to defects in polymerase POLE (**Additional file 1: Figure S2B**). The survival analysis revealed that the PFS (**Figure S2C**) and OS (**Additional file 1:** **Figure S2D**) had no significant difference among the three signature groups.

### 3.3 Co-occurrence and mutual exclusivity in the FPHYP cohort

**Figure 5:**
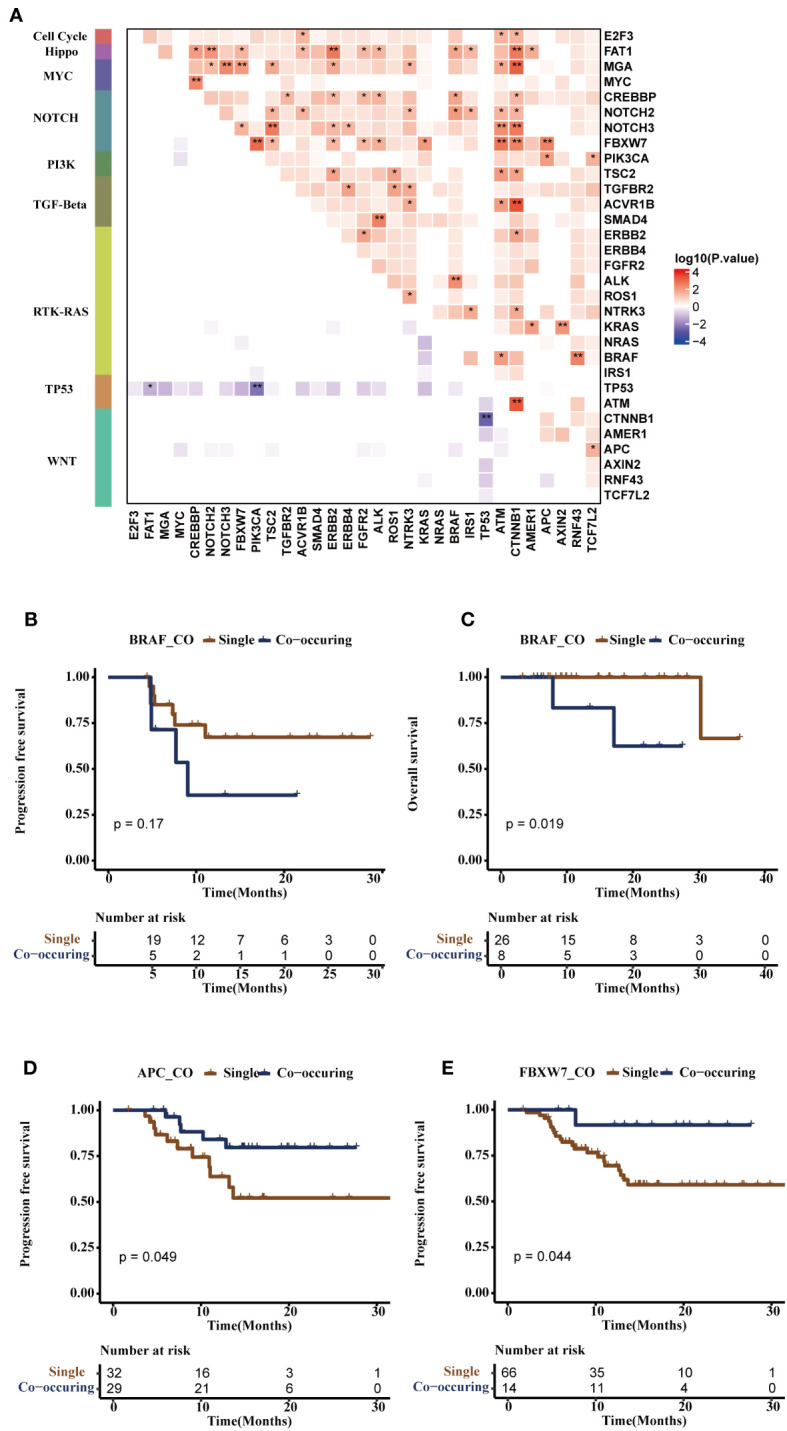
Co-mutational and mutual-exclusive features of the FPHYP cohort. **(A)** The correlation plot depicted the co-occurrence and mutual exclusivity relationships of gene alterations within oncogenic signaling pathways. **(B, C)** Kaplan-Meier survival analysis was conducted for single-gene mutations and co-occurring gene pairs (*BRAF*/*ALK*, *BRAF*/*NOCH2*, *BRAF*/*CREBBP* or *BRAF*/*FAT1*) on PFS **(B)** and OS **(C)** outcome in CRC patients. **(D)** The Kaplan-Meier survival analysis of single-gene *APC* mutation and co-occurring gene pairs (*APC*/*PIK3CA*, or *APC*/*FBXW7*) on PFS outcome in CRC patients. **(E)** The Kaplan-Meier survival analysis of single-gene mutation and co-occurring gene pairs (*FBXW7*/*APC*, *FBXW7*/*CTNNB1*, *FBXW7*/*ATM*, *FBXW7*/*KRAS*, *FBXW7*/*ALK*, *FBXW7*/*FGFR2*, *FBXW7*/*ERBB2*, *FBXW7*/*TSC2*, or *FBXW7*/*PIK3CA*) on PFS in CRC patients. *p < 0.05, **p < 0.01. PFS, progression-free survival; OS, overall survival.

To examine the dynamic changes and the function of gene altered pairs during tumorigenesis and development, we performed an analysis with a specific focus on detecting noteworthy patterns of mutation genes that co-occur or are mutually exclusive. We constructed a genomic alteration map to delve into oncogenic interdependencies, resulting in the identification of 80 significant co-occurring gene pairs and three pairs showing mutual exclusivity (p ≤ 0.05, **Additional file 2: Table S4**). Notably, we found distinct mutually exclusive relationships within the TP53 and WNT pathway, while multiple co-occurring alterations per sample were observed in pathways of RTK-RAS, NOTCH, WNT, PI3K, TGF-Beta, MYC, and PI3K (**Figure 5A**).

Additionally, we examined the noteworthy occurrence and exclusion of mutation pairs in non-metastatic and metastatic CRC to assess the dynamic alterations and functions of gene altered pairs throughout tumor progression. *MYC*/*CREBBP*, *TP53*/*APC* co-mutation, mutually exclusive *TP53*/*ATM*, and *TP53*/*BRAF* were more occurrence in mCRC, while the co-occurring pairs *BRAF*/*ALK* (*NOTCH2*, *CREBBP*, or *FAT1*) and *FBXW7*/*APC* (*CTNNB1*, *ATM*, *KRAS*, *ALK*, *ERBB2*, *TSC2*, or *PIK3CA*) were observed in non-mCRC. Mutually-exclusive *TP53*/*PIK3CA* was observed in non-mCRC and mCRC (**Additional file 1: Figures S3A, B**). Subsequently, the survival analysis showed *BRAF*/*ALK* (*NOTCH2*, *CREBBP*, or *FAT1*) were associated with shorter PFS (p = 0.17, **Figure 5B**) and OS (p = 0.019, **Figure 5C**) in CRC patients. *APC*/*PIK3CA* (or *FBXW7*) co-occurring pairs were found to be associated with longer PFS (p = 0.049, **Figure 5D**), consistent with previous research (28). On the other hand, *FBXW7/CTNNB1* (*ATM*, *KRAS*, *ALK*, *ERBB2*, *AP*C, *TSC2*, or *PIK3CA*) co-occurring pairs were also associated with longer PFS (p = 0.044, **Figure 5E**), although further studies are needed to validate this finding.

### 3.4 Germline alterations profile and fusion genes of FPHYP cohort

**Figure 6:**
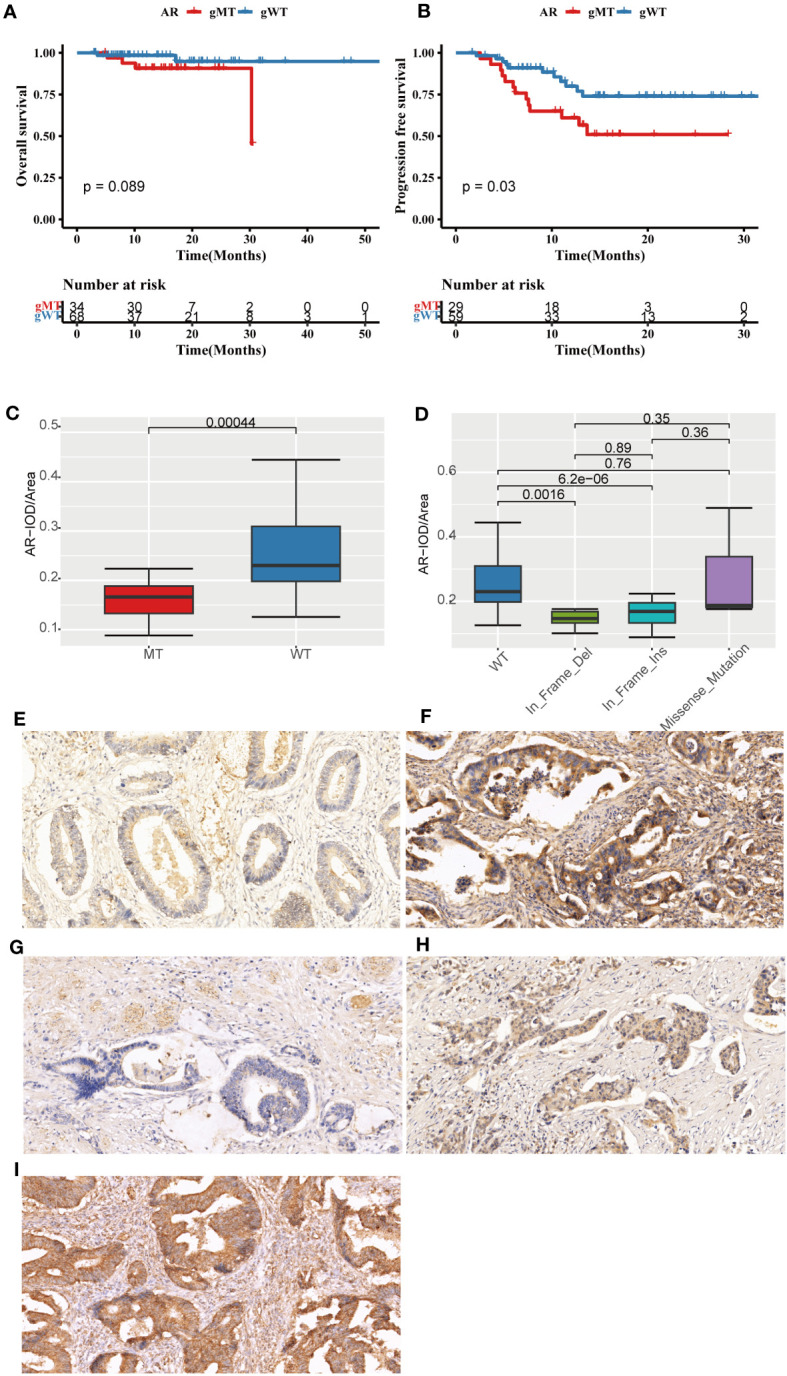
The prognostic significance and immunohistochemical analysis of AR in the FPHYP cohort. **(A, B)** The Kaplan-Meier survival analysis for OS **(A)** and PFS **(B)** between CRC cases with *AR* germline variant group (gMT) and without *AR* germline variant group (gWT). **(C)** Comparison of the IOD/Area value between gMT and gWT groups. **(D)** Comparison of the IOD/Area value among different mutation types of WT, In_Frame_Del, In_Frame_Ins, and Missense_Mutation groups. **(E, F)** AR protein expression original field acquired from tissue sections (magnification, 200x) of gMT **(E)** and gWT **(F)** groups. **(G–I)** AR protein expression original area obtained from tissue sections (magnification, 200x) of In_Frame_Del **(G)**, In_Frame_Ins **(H)**, and Missense_Mutation **(I)** groups. PFS, progression-free survival; OS, overall survival; gWT, with *AR* germline variant group; gWT, without germline variant group. IOD, cumulative optical density.

In this FPHYP cohort, the top frequently germline mutated genes were *AR* (34%), *FAT1* (17%), *ZFHX3* (17%), *LRP1B* (15%), *KMT2A* (12%), *KMT2D* (12%), *CIC* (11%), *KMT2C* (11%), and *BRCA2* (11%), respectively (**Additional file 1: Figure S4A**). In germline evaluation of 111 CRC patients, 1289 mutations were found, of which nine were identified as pathogenic or likely pathogenic (**Table 2**). Furthermore, germline *AR* variant carriers (gMT) were associated with shorter OS (p = 0.089; **Figure 6A**) and PFS (p = 0.03; **Figure 6B**) than those detected without germline *AR* mutation cases (gWT) in FPHYP cohort. Thirty-seven patients with 42 germline AR mutations were detected (**Additional file 1: Figure S4B**, **Additional file 2: Table S5**).

**Table 2:** The germline pathogenic/likely pathogenic alterations in the FPHYP cohort.

Sample ID	Chromosome	Start_Position	End_Position	Reference_Allele	Variant_Allele	Hugo_Symbol	DDR pathway	Variant_Classification	Variant Detail	Variant Allele Frequency	rs number (SNP150)	rs number (SNP138)	ClinVar Annotation
Patient 23	chr19	15297982	15297982	g	a	NOTCH3	/	nonsynonymous SNV	NOTCH3:NM_000435:exon11:c.1774C>T:p.R592C	47.57%	rs764148985	.	Likely_pathogenic
Patient 24	chr6	162206825	162206825	c	g	PRKN	/	nonsynonymous SNV	PRKN : NM_004562:exon7:c.850G>C:p.G284R	50.46%	rs751037529	.	Pathogenic
Patient 71	chr7	6026658	6026658	t	a	PMS2	MMR	stop gain	PMS2:NM_000535:exon11:c.1738A>T:p.K580*	53.23%	rs267608169	rs267608169	pathogenic
Patient 72	chr7	6045664	6045664	t	c	PMS2	MMR	splicing	PMS2:NM_000535:exon2:c.24-2A>G	48.14%	.	.	likely_pathogenic
Patient 84	chr10	101560288	101560288	c	t	ABCC2	/	nonsynonymous SNV	ABCC2:NM_000392:exon9:c.1177C>T:p.R393W	47.35%	rs777902199	.	likely_pathogenic
Patient 91	chr11	94169066	94169066	c	a	MRE11	/	splicing	MRE11:NM_005591:exon18:c.1927-1G>T	50.20%	.	.	likely_pathogenic
Patient 91	chr2	234681059	234681059	t	g	UGT1A1	/	nonsynonymous SNV	UGT1A1:NM_000463:exon5:c.1456T>G:p.Y486D	46.95%	rs34993780	rs34993780	pathogenic/likely_pathogenic
Patient 97	chr13	32914210	32914211	ct	–	BRCA2	HR	frameshift deletion	BRCA2:NM_000059:exon11:c.5722_5723del:p.L1908fs	49.88%	rs80359530	rs80359530	pathogenic
Patient 98	chr1	47748039	47748039	c	g	STIL	/	nonsynonymous SNV	STIL : NM_003035:exon11:c.1226G>C:p.S409T	49.57%	rs369348360	rs369348360	likely_pathogenic

The immunohistochemical (IHC) detection showed that the protein expressions of AR were much lower in the gMT group than those in the gWT group of the FPHYP cohort (**Figures 6E, F**). The IOD/Area analysis for IHC detection of AR (p < 0.001) in gMT and gWT groups was shown (n=64) in **Figure 6C**. Meanwhile, the AR protein expression level was associated with the germline alteration type, highest in cases with nonsynonymous SNV and lowest in those with in-frameshift deletion (**Figures 6G–I**).

Eight fusion genes were identified in seven patients with CRC. These fusion genes include *RPSAP52*-*HMGA2*, *KHDRBS3-FGFR1*(exon3), *KHDRBS3*-*FGFR1*(exon4), *TPR*-*NTRK1*, *PTPRK*-*RSPO3*, *PTPRK*(exon7)-*RSPO3*, and *PTPRK*(exon1)-*RSPO3* (**Additional file 2: Table S6**). Among these, *RPSAP52*-*HMGA2* and two *KHDRBS3*-*FGFR1* were newly discovered novel fusions.

### 3.5 Development and verification of a gene signature based on mutations

**Figure 7:**
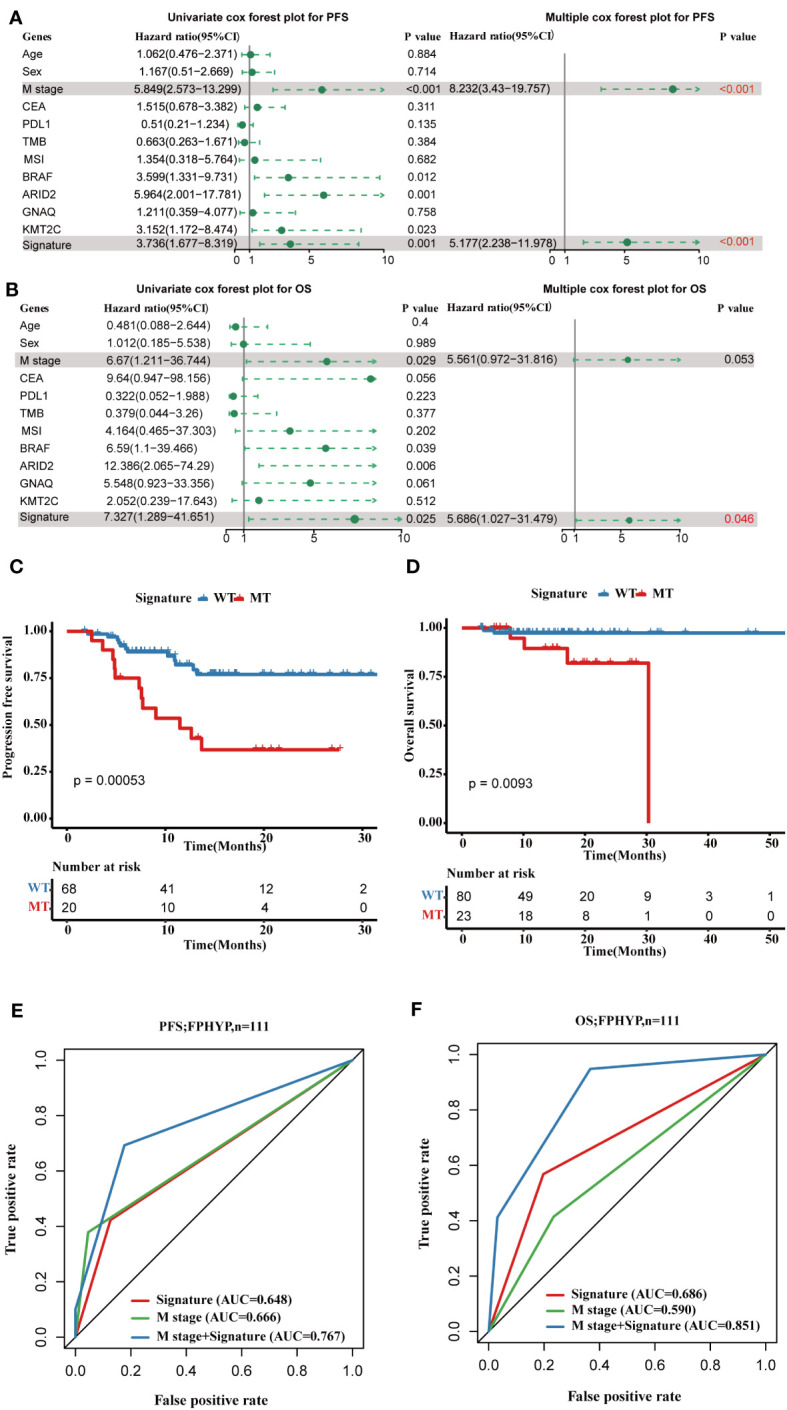
Construction of a four-gene mutation signature prediction disease progression and prognosis in FPHYP cohort. **(A, B)** Univariate and multivariate analyses were performed to assess the impact of clinicopathological features, individual somatic gene mutations, and the four-gene mutation signature on PFS **(A)** and OS **(B)** in CRC. **(C)** The Kaplan-Meier survival analysis for PFS in CRC patients between the four-gene combined MT and WT groups based on *BRAF*, *ARID2*, *KMT2C*, and *GNAQ* mutation status. **(D)** The Kaplan-Meier survival analysis for OS in CRC cases between the four-gene combined MT and WT groups based on *BRAF*, *ARID2*, *KMT2C*, and *GNAQ* mutation status. **(E, F)** ROC curves for PFS **(E)** and OS **(F)** that dependent on time were generated to evaluate the prognostic model’s performance, which is based on the gene mutation status within the FPHYP cohort. PFS, progression-free survival; OS, overall survival; MT, mutation type; WT, wiled type; ROC, receiver operating characteristic.

To explore the prognostic significance of the mutation genes, we constructed a gene set signature based on the gene mutation status and prognostic significance. According to the relationship of single-gene mutation prognostic significance with PFS and OS, four genes(*BRAF*, *ARID2*, *KMT2C*, and *GNAQ*) were filtered to construct the four-gene mutation signature. Univariate and multivariate Cox regression analyses showed that the four-gene mutation signature correlated with PFS (Univariate: hazard ratio, 3.736; 95% CI, 1.677–8.319; p = 0.001; multivariate: hazard ratio, 5.177; 95% CI, 2.238–11.978; p = 0.00012; **Figure 7A**) and OS ((Univariate: hazard ratio, 7.327; 95% CI, 1.289–41.651; p = 0.025; multivariate: hazard ratio, 5.686; 95% CI, 1.027–31.479; p = 0.0465; **Figure 7B**). Patients in the MT group of four-gene (with at least one mutation of *BRAF*, *ARID2*, *KMT2C*, or *GNAQ*) had a shorter PFS (p = 0.00053; **Figure 7C**) and OS (p = 0.0093; **Figure 7D**) than those in the WT group of four-gene (without any mutation of *BRAF*, *ARID2*, *KMT2C*, and *GNAQ*). The AUC of PFS and OS of the four-gene mutation signature in the FPHYP cohort were 0.648 and 0.686 (**Figures 7E, F**). When combined metastases status and signature the AUC were 0.767 for PFS and 0.851 for OS, much better than the single signature.

**Figure 8:**
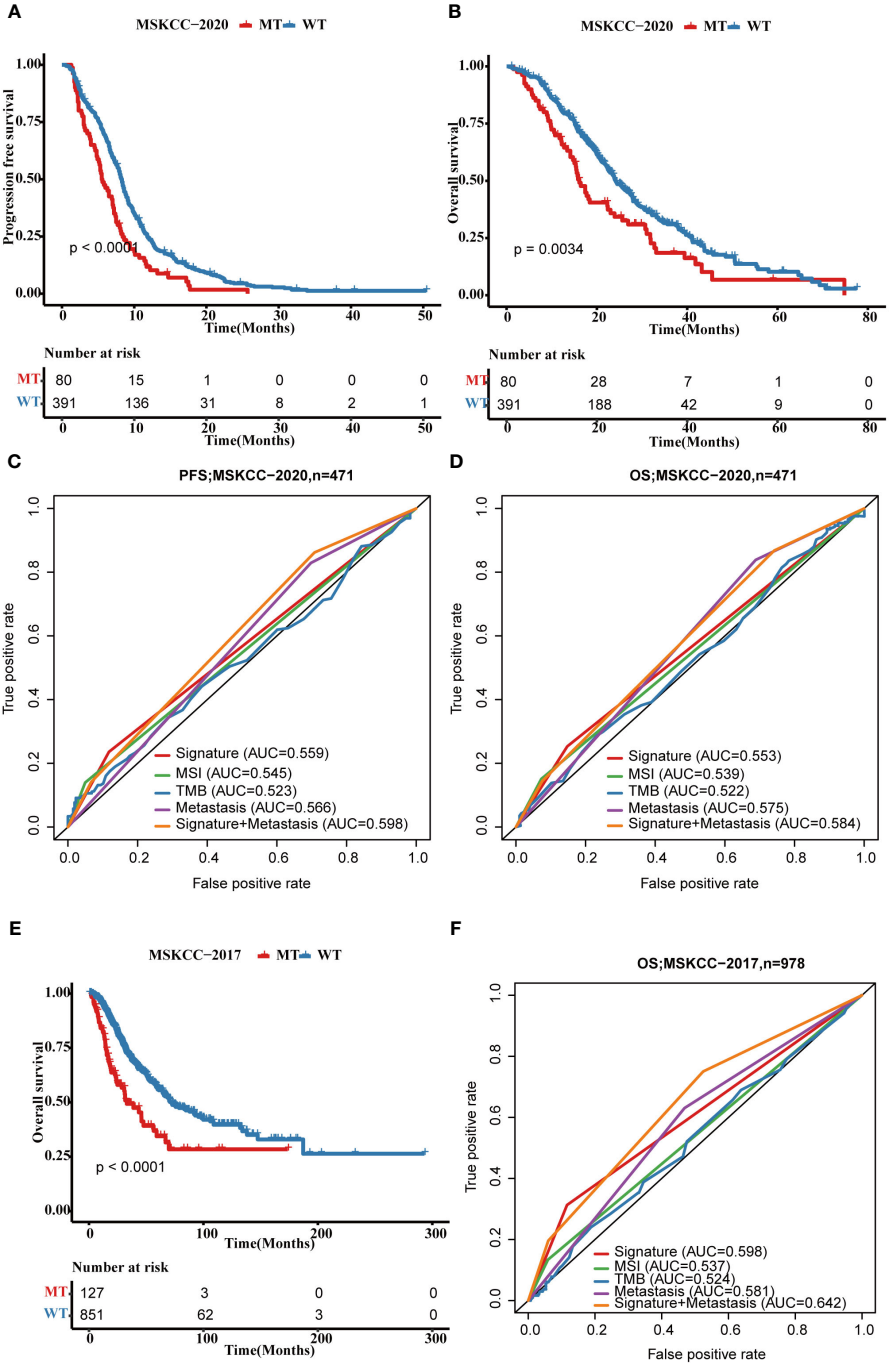
Validation of the four-gene mutation signature in MSKCC cohorts. **(A)** The Kaplan-Meier survival analysis of four-gene mutation signature on PFS outcome MSKCC-2020 cohort patients. **(B)** The Kaplan-Meier survival analysis of the four-gene mutation signature on OS outcome of MSKCC-2020 cohort patients. **(C, D)** ROC curves that dependent on time were generated to evaluate the prognostic model’s performance for PFS **(C)** and OS **(D)**, which is based on the gene mutation status within of the four-gene mutation signature and clinical characteristics in the MSKCC-2020 cohort. **(E)** The Kaplan-Meier survival analysis of four-gene signature on OS outcome MSKCC-2017 cohort patients. **(F)** ROC curves that dependent on time were generated to evaluate the prognostic model’s performance, which is based on the gene mutation status within MSKCC-2017 cohort patients. PFS, progression-free survival; OS, overall survival; MT, mutation type; WT, wiled type; ROC, receiver operating characteristic.

Subsequently, we validated the four-gene mutation signature in MSKCC-2017 and MSKCC-2020 cohorts. Patients in the MT group also showed a shorter PFS (MSKCC-2020: p < 0.0001; **Figure 8A**) and OS (MSKCC-2020: p = 0.0034; **Figure 8B**; MSKCC-2017 cohort: p < 0.0001; **Figure 8E**) than those in the WT group in two validated cohorts, which is consistent with those findings in the FPHYP cohort. The AUC of the mutation signature in the validation cohorts were 0.559 for PFS and 0.553 for OS in the MSKCC-2020 cohort (**Figures 8C, D**) and 0.598 for OS in the MSKCC-2017 cohort (**Figure 8F**), respectively. When combined metastases status and signature the AUC were 0.598 for PFS and 0.584 for OS in the MSKCC-2020 cohort, and 0.642 for OS in the MSKCC-2017 cohort, much better than the single signature, which is consistent with those in the FPHYP cohort. The above findings identified that the four-gene mutation signature was an independent predictor of prognosis.

Additionally, to explore the molecular mechanisms altering clinical characteristics, we evaluated the association of the four-gene mutation signature with other clinical factors in CRC. MSI and TMB are important indicators with a close relationship to immune therapy in CRC (29). The carbohydrate antigen19-9 (CA19-9) and carcinoembryonic antigen (CEA) are representative serum tumor markers commonly used in clinical practice for CRC patients (30). Metastasis plays a crucial role in determining patient prognosis and survival in CRC. Around 20% of CRC patients already have metastases at diagnosis, and up to 50% of those initially diagnosed with localized disease will eventually develop metastases (31). The patients with MT four-gene mutation signature and TMB-high had worse survival than those with WT four-gene mutation signature and TMB-high, and patients with MT four-gene mutation signature and TMB-low had worse survival than those with WT four-gene mutation signature and low TMB-low (**Additional file 1: Figure S5A**). Similar results were obtained in the CEA, CA19-9, tumor size, metastasis status, and MSI stratifying groups (**Additional file 1: Figures S5B–F**).

### 3.6 Development and evaluation of the nomogram and validation of the protein expression

**Figure 9:**
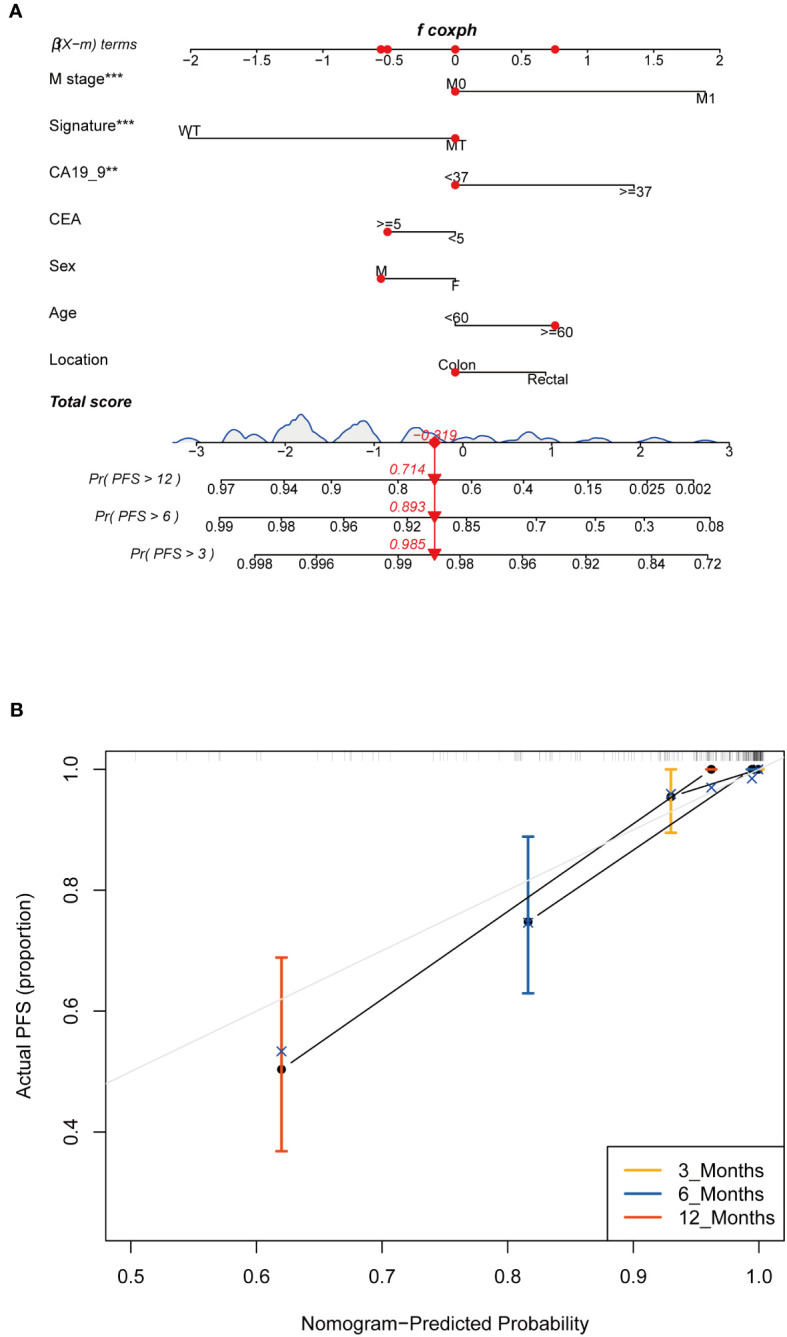
Construction and assessment of the prognostic nomogram with four-gene mutation signature as one of the parameters. **(A)** A prognostic nomogram was constructed using the four-gene mutation signature and clinicopathological factors to predict 3-, 6-, and 12-month survival rates for PFS of CRC patients. **(B)** Calibration curves showing the probability of 3-, 6-, and 12-month survival rates for PFS in the FPHYP cohort. PFS, progression-free survival. ** Indicates p<0.01, *** Indicates P<0.001.

In the FPHYP cohort, we developed nomograms to predict the PFS using multivariable Cox and stepwise regression analyses. These nomograms were designed to incorporate three crucial factors: age, tumor stage, and a four-gene mutation signature (**Figure 9A**). To ensure the reliability and accuracy of the nomograms, we conducted calibration curves to validate their performance in predicting survival rates at different time intervals. For PFS, the calibration curves were assessed at 3-, 6-, and 12 months (**Figure 9B**). The calibration curves demonstrate the nomograms’ ability to provide accurate predictions of survival rates over these specific time frames.

**Figure 10:**
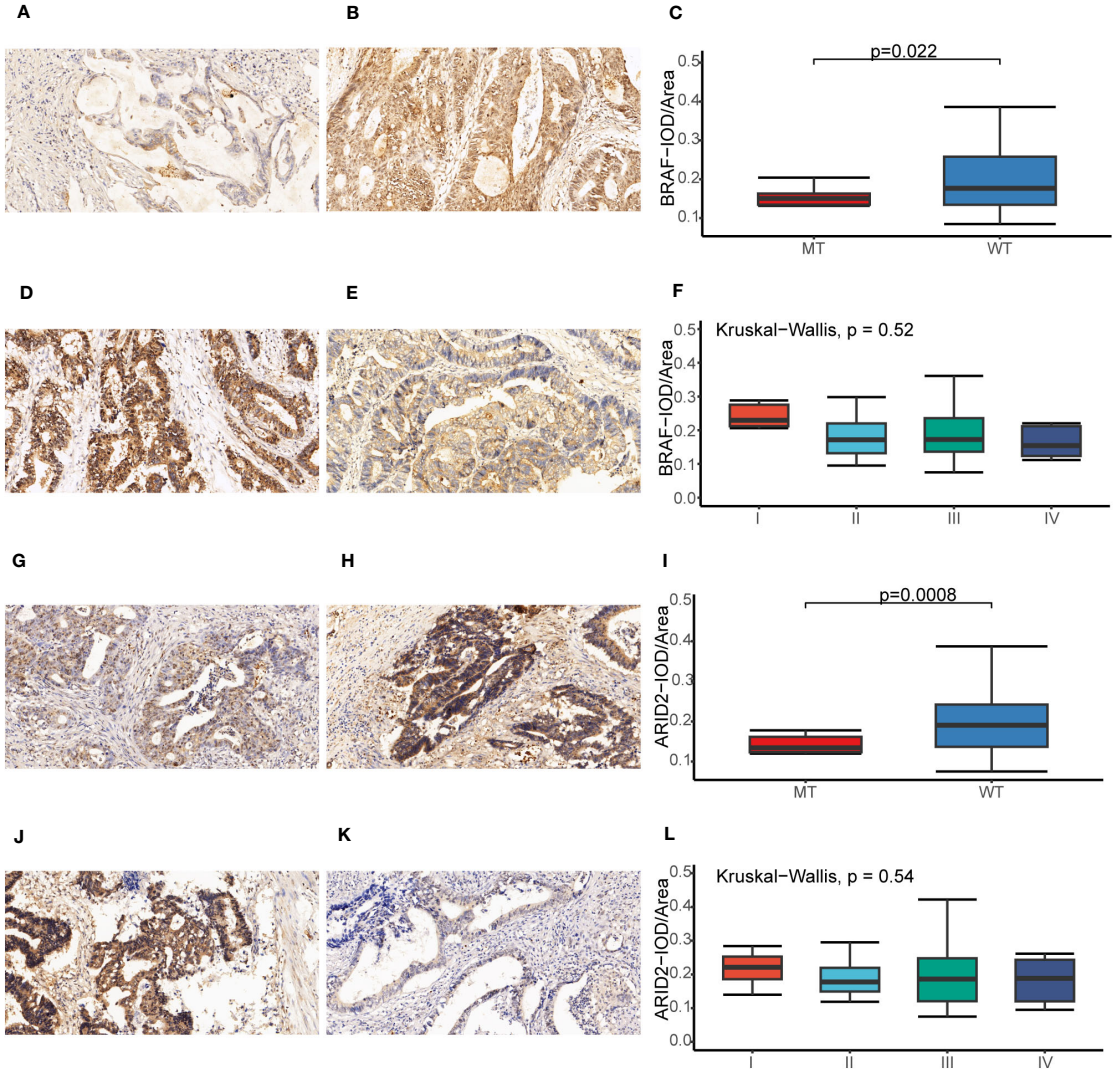
Immunohistochemical analysis of BRAF and ARID2 in CRC. **(A, B)** The BRAF expression original field was acquired from tissue sections (magnification, 200x) of the *BRAF*-MT **(A)** and *BRAF*-WT **(B)** groups. **(C)** Comparison of the IOD/Area value between *BRAF*-MT and *BRAF*-WT groups. **(D, E)** The BRAF expression original field was acquired from tissue sections (magnification, 200x) of stage I **(D)** and stage IV **(E)** groups. **(F)** Comparison of the IOD/Area value between stage I and IV groups. **(G, H)** The ARID2 expression original field was acquired from tissue sections (magnification, 200x) of the *ARID2*-MT **(G)** and *ARID2*-WT **(H)** groups. **(I)** Comparison of the IOD/Area value between *ARID2*-MT and *ARID2*-WT groups. **(J, K)** The ARID2 expression original field was acquired from tissue sections (magnification, 200x) of stage I **(J)** and stage IV **(K)** groups. **(L)** Comparison of the IOD/Area value between stage I and IV groups. MT, mutation type; WT, wiled type; IOD, cumulative optical density.

Our study utilized the IHC method to assess the protein expression levels of BRAF and ARID2 in CRC tissues. The positive-staining density, measured as IOD/Area, was quantified using Image Pro Plus 6.0 software (32). Our findings revealed a notable correlation between the protein expression levels of BRAF and ARID2 and the genetic status of these two genes in CRC. Precisely, in CRC cases with a mutated (MT) form of BRAF or ARID2, the protein expression levels of BRAF or ARID2 were significantly lower compared to cases with the wild-type (WT) form (p = 0.022, **Figures 10A–C** for BRAF; p = 0.0008, **Figures 10G–I** for ARID2). Furthermore, when considering tumor stage, we observed that higher stages of CRC were associated with decreased expression levels of BRAF and ARID2 proteins (p = 0.52, **Figures 10D–F** for BRAF; p = 0.54, **Figures 10J–L** for ARID2). This suggests that reduced expression of these proteins may be indicative of advanced tumor stages. Intriguingly, we also found differences in protein expression levels between rectal and colon tumors. Specifically, BRAF protein expression was higher in rectal tumors compared to colon tumors (p =0.077, **Additional file 1: Figures S6A–C**). Moreover, the expression level of ARID2 protein was found to have a significant correlation with the gender of CRC patients. It was higher in male patients compared to female patients (p =0.083, **Additional file 1: Figures S6D–F**).

## 4 Discussion

In this study, we performed NGS sequencing on 111 CRC tissue samples and their matched peripheral blood samples using an ultradeep 599-gene panel, generating a large CRC cohort with comprehensive genomic and clinical data. Our rich clinical dataset, including treatment information, metastatic status, serum marker protein expression level, progression-free survival, and overall survival follow-up, provides an important resource for exploring CRC genetic risks, identifying additional prognostic biomarkers, and predicting therapeutic response/resistance. Prominent treatment protocols employed for CRC primarily involve fluoropyrimidines in conjunction with either oxaliplatin or irinotecan, often incorporating targeted agents like bevacizumab, cetuximab, or panitumumab (33). Notably, median OS for CRC patients has surpassed 33 months in phase 3 trials (34). Immunotherapy, specifically immune checkpoint inhibitors, has emerged as a significant area of focus in treating colorectal cancer. The FDA has granted approval for pembrolizumab, an anti-PD-1 monoclonal antibody, to be used in treating TMB-high solid tumors in adults and children. This approval marks a crucial step in pursuing more effective therapies for colorectal cancer patients (35).

We compared the genomic alterations and clinicopathological characteristics between our FPHYP cohort and two cohorts from MSKCC. *ATM*, *ALK*, *GNAS*, *GNAQ*, *E2F3*, *ERCC3*, *CDK8*, *STAT5B*, and *CHEK1* genes were significantly highly enriched in the FPHYP cohort compared to two MSKCC cohorts. Our cohort showed the highest proportion of males, the lowest ratio of mCRC patients, the highest proportion of rectal cancer cases, and most TMB-high patients. From 1990 to 2019, the number of newly diagnosed cases of colorectal cancer worldwide has more than doubled, rising from 842,098 cases in 1990 to 2.17 million cases in 2019. Among the genders, males experienced a higher incidence rate, death toll, and DALYs (Disability-Adjusted Life Years) related to colon and rectal cancer than females. China had the highest number of newly diagnosed and deceased cases (36). The alterations were primarily enriched in HIPPO, TP53, and NOCTH pathways in the FPHYP cohort. Furthermore, the incidence of alterations in the RTK-RAS and WNT pathways was higher in the mCRC group compared to the non-metastatic group in FPHYP cohort.

By examining the associations between gene mutations and PFS/OS, we discovered that *BRAF*, *ARID2*, *KMT2C*, and *GANQ* mutations are associated with worse clinical outcomes. Mutations in the *BRAF* gene lead to its constitutive activation, causing the RAS/RAF/MEK/ERK signaling pathway to be constantly “on,” even without external signals. This abnormal pathway activation has been linked to various cancers, including melanoma, CRC, thyroid cancer, and others. Notably, in mCRC patients, *BRAF* mutations have been identified as significant negative prognostic markers (6). On the other hand, *ARID2* is considered a tumor suppressor gene that regulates cell growth, differentiation, and apoptosis. Mutations or alterations in the *ARID2* gene have been implicated in different cancers, such as hepatocellular carcinoma, ovarian cancer, colorectal cancer, and lung adenocarcinoma. Loss of ARID2 function due to these genetic changes can lead to dysregulation of gene expression, contributing to cancer development and progression. Researchers have found that during the occurrence and development of lung adenocarcinoma in humans and mice, the expression level of ARID2 gradually decreases (37). Similarly, *KMT2C* is a critical gene involved in various biological processes, including embryonic development, cell differentiation, and cell cycle regulation. It functions as a tumor suppressor, helping to control cell growth and prevent tumor formation. Mutations in the *KMT2C* gene have been found in gastric, CRC, and endometrial cancer, indicating its role in tumor development (38). In colorectal cancer, *KMT2C* loss-of-function mutations (LOF) are connection with increased genomic instability. Additionally, these LOF mutations are linked to decreased regulatory T cells and increased CD8+ T cells, activated NK cells, M1 macrophages, and M2 macrophages (27). Notably, in CRC, *KMT2C* LOF mutations are correlated with extended overall survival and better clinical responses to PD-1 immunotherapy, particularly in Chinese patients (39). Furthermore, *GNAQ* gene mutations have been identified in ocular melanoma, where they play a crucial role in tumor development and progression (40). In conclusion, these genetic findings highlight the importance of understanding the roles of *BRAF*, *ARID2*, *KMT2C*, and *GNAQ* in different cancer types, shedding light on potential therapeutic targets and predictive markers for cancer treatment strategies.

A study discovered that 16% of CRC showed hypermutation. Among these hypermutated tumors, approximately three-quarters demonstrated MSI-H, which was typically associated with MLH1 hypermethylation and inactivation. The remaining one-quarter of hypermutated tumors had somatic mutations in mismatch repair genes and polymerase (POLE) (14). Our study identified two patients with hypermutation (TMB >100 mut/MB). The TMB values were 130.13 for Patient 39 and 563.57 for Patient 46. The two patients harbored 135 and 696 somatic mutations, respectively. At the same time, we also observed multiple genes with both somatic and germline mutations in these two patients, including *HIST3H3*, *KEL*, *ADGRA2*, *SYK*, *POLE*, *PALB2*, *TOP2A*, *SMARCA4*, *ARID1B*, *NOTCH2*, and *MSH6* (**Additional file 2: Table S7**). In 1971, American medical expert Alfred G. Knudson proposed the “Two-hit Hypothesis,” which states that “two hits” to tumor suppressor genes are necessary for the development of cancer (41). This hypothesis suggests that the inactivation or loss of both copies of a tumor suppressor gene is a critical event in cancer progression. The first hit may be inherited, affecting one copy of the gene in the germline (germline mutation). In contrast, the second hit involves the somatic mutation or loss of the remaining functional copy in a specific cell or tissue. This leads to the loss of tumor suppressor gene function and contributes to cancer development. Fortunately, the germline mutations in both patients have been assessed and determined not to be classified as pathogenic or likely to cause disease.

In a large cohort from The Cancer Genome Atlas (TCGA), researchers found significant associations between germline genomic patterns and COSMIC signatures, indicating the role of heredity in cancer development. This study highlights the importance of genetic factors in cancer causation (42). CRC in first-degree relatives with Lynch syndrome has been linked to DNA repair defects in their germ cells. These defects impair the resurrection of DNA damage, resulting in the accumulation of genetic alterations that contribute to the development of colon cancer (43). Germline alterations classified as pathogenic or likely pathogenic (**Table 2**) within the DNA damage response (DDR) pathway represent encouraging candidates for targeted treatment with PARP inhibitors in CRC (44). The androgen receptor (AR) belongs to the nuclear receptor super-family and is a steroid receptor. It acts as a transcription factor, regulating gene expression in eukaryotic organisms, and plays a crucial role in developing and maintaining various systems, including reproduction, skeletal muscles, cardiovascular, nervous, immune, and hematopoietic systems (45). Previous researches show that germline mutations of *AR* gene are associated with the incidence of prostate cancer (PCa) (46) and the development of breast cancer (47).

The co-survival analysis found *AR* germline mutations associated with shorter PFS and OS. Further analysis revealed 52.3% of *AR* germline mutations were non-frameshift insertions, 33.3% were non-frameshift deletions, and 13.3% were nonsynonymous single nucleotide variants (SNVs). To investigate the impact of *AR* germline mutations on protein expression levels, we conducted IHC experiments for validation. The findings revealed a significant correlation between AR protein expression levels and AR germline mutation status. Moreover, the results also indicated a correlation between AR protein expression levels and the specific type of mutation present in the germline (**Figures 6D, G–I**). A recent report highlighted the potential of membrane androgen receptors (ARs) in colon tumors to induce apoptosis in cancerous cells (48). A study suggests that colonic tumor cells could upregulate pro-apoptotic pathways through an AR-dependent mechanism, resulting in tumor regression. Furthermore, the presence of ARs might offer new avenues for pharmacological therapy targeting colorectal neoplasms (49). Based on the above findings, we consider *AR* germline mutations as a risk factor in colorectal cancer, although further research is needed to validate this discovery due to limited research on *AR* germline mutations in colon cancer.

In 2022, Long J et al. performed a comprehensive genomic analysis across the pan-cancer to identify a potent signature based on a specific set of mutation genes. This signature was developed to predict the clinical benefits of immune checkpoint inhibitor (ICI) treatment in patients (50). Our study demonstrated that *BRAF*, *ARID2*, *KMT2C*, and *GANQ* mutations associated with worse clinical outcomes. Subsequently, we constructed a mutation-based signature based on these four genes to predict the prognosis of CRC. The univariate and multivariate Cox regression analyses revealed the four-gene mutation signature was an independent predictor of prognosis. We also validated this signature in two MSKCC cohorts. The results were consistent with those in the FPHYP cohort. The AUC of the FPHYP cohort was a little better than in the MSKCC cohorts. The reason may be the differences in patient composition. In two MSKCC cohorts, the composition of metastatic CRCs was much higher than those in FPHYP cohort.

Finally, we established a nomogram to predict the PFS of CRC based on the four-gene mutation signature and the clinicopathological characteristics. By integrating age, stage, and the four-gene mutation signature into the nomograms, we aimed to offer a comprehensive and reliable tool for predicting PFS and OS in the FPHYP cohort. These nomograms have the potential to assist clinicians in making informed decisions and improving patient outcomes by identifying individuals at higher risk of disease progression and poor OS.

To explore the correlation of protein expression level with genetic status and clinicopathological characteristics, BRAF and ARID2 were detected by IHC. Our study demonstrates significant associations between the protein expression levels of BRAF and ARID2 and the genetic status of these genes in CRC. Additionally, we observed differences in protein expression levels based on tumor stage, tumor location (rectal vs. colon), and patient gender. These findings contribute to a better understanding of the molecular characteristics of CRC and may have implications for future diagnostic and therapeutic strategies.

Moreover, the incorporation of mutational signatures derived from multiple gene mutations and their association with CRC prognosis holds significant clinical relevance. This comprehensive approach improves risk assessment, allowing for a more precise evaluation of an individual’s disease susceptibility and facilitating personalized prevention strategies. It also proves valuable in the accurate diagnosis of complex genetic conditions, particularly in guiding treatment selection, a pivotal component of precision medicine, notably in the field of oncology. This signature also contributes to prognosis evaluation, streamlines the design of clinical trials, and aids in drug development by uncovering novel therapeutic targets. Overall, the mutation-based signature has the potential to transform disease management and enhance patient outcomes within the realm of clinical practice.

Our study has several limitations that should be considered. Firstly, the sample size of our cohort is relatively small. Additionally, the computation of TMB and MSI were based on targeted panel sequencing. Furthermore, the follow-up time for the HPHYP cohort, particularly in cases of primary CRC, is relatively short. However, despite these limitations, this study successfully identified the clinical practicability and significance of extensive prospective sequencing. Our in-depth examination of clinical and molecular characteristics enabled the four-gene mutation signature with predictive capabilities for prognosis.

## 5 Conclusion

Our study has successfully identified a four-gene mutation signature, the first comprehensive genomic marker for predicting prognosis in CRC. This research is also a large project focusing on discovering prognostic models for cancer patients who have undergone chemotherapy or a combination of chemotherapy and targeted therapy, such as bevacizumab or cetuximab. Integrating the four-gene mutation signature with clinicopathological characteristics into a nomogram can assist clinicians in selecting the most appropriate treatment approach for individual patients. Additionally, our study has validated the protein expression of *AR*, *BRAF*, and *ARID2*, shedding light on how mutations impact protein expression. Overall, this work introduces a novel signature that holds promise in predicting CRC recurrence and prognosis, offering crucial insights to clinicians in making informed treatment decisions.

## Data availability statement

All data and material from this study are available. The data of 111 CRC patients in FPHYP cohort can be found in the article/Supplementary Material. The publicly available datasets data of genome sequencing, survival, and clinical information of Metastatic Colorectal Cancer (MSK, Cancer Cell 2018) and Colorectal Cancer (MSK, Gastroenterology 2020) from the Memorial Sloan Kettering Cancer Center (MSKCC) database were downloaded from cBioPortal (http://www.cbioportal.org).

## Ethics statement

All samples were approved and authorized by the First People’s Hospital of Yunnan Province Ethics Committee, and all participants provided informed consent. The studies were conducted in accordance with the local legislation and institutional requirements. Written informed consent for participation in this study was provided by the participants’ legal guardians/next of kin.

## Author contributions

JY: Conceptualization, Project administration, Writing – original draft. SZ: Conceptualization, Resources, Writing – original draft. JSu: Conceptualization, Formal Analysis, Writing – original draft. SL: Methodology, Visualization, Writing – review & editing. ZW: Data curation, Investigation, Writing – review & editing. WM: Data curation, Investigation, Writing – review & editing. MT: Data curation, Investigation, Writing – review & editing. JW: Data curation, Investigation, Writing – review & editing. EM: Data curation, Investigation, Writing – review & editing. LH: Formal Analysis, Validation, Writing – review & editing. ML: Formal Analysis, Validation, Writing – review & editing. JZ: Formal Analysis, Validation, Writing – review & editing. LC: Formal Analysis, Validation, Writing – review & editing. JSh: Conceptualization, Supervision, Writing – review & editing. YS: Conceptualization, Supervision, Writing – review & editing.
