# Supplementary material for: Comprehensive genomic profiling reveals prognostic signatures and insights into the molecular landscape of colorectal cancer
Source: Front Oncol. 2023 Nov 13;13:1285508. doi: 10.3389/fonc.2023.1285508 (PMC10680082; doi:10.3389/fonc.2023.1285508)
Supplement: Supplementary file 1 [file DataSheet_1.docx]

**
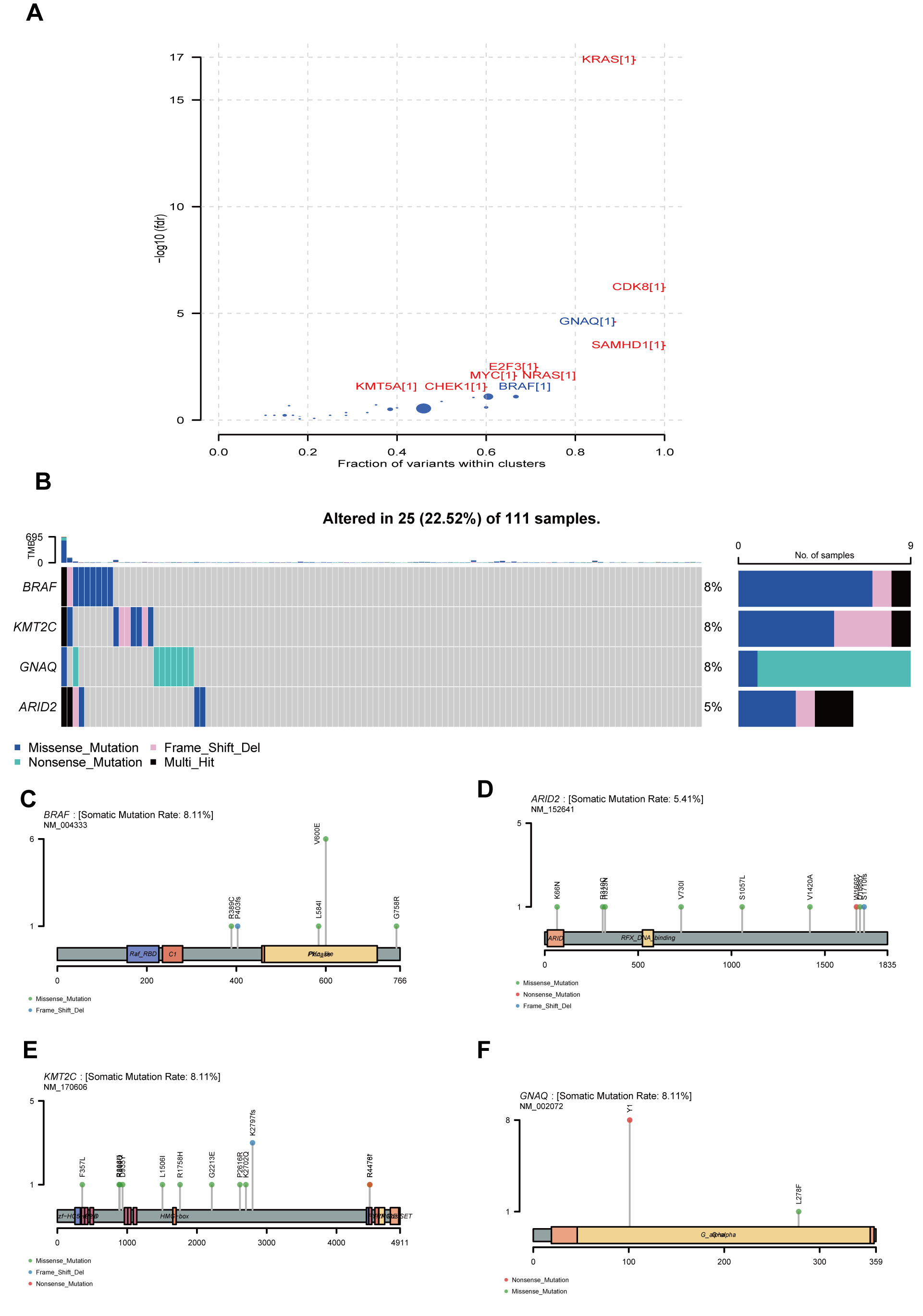
**

**Supplementary Figure 1**. **The four driver genes mutation characteristics and** **mutation signature of the FPHYP cohort.** (**A**) The driver mutations and significantly mutated genes of CRC cases. (**B**) The mutation landscape of four prognostic genes. (**C-F**) Lollipop plots of the somatic variant in *BRAF* (**C**), *ARID2* (**D**), *KMT2C* (**E**), and *GNAQ* (**F**) genes.


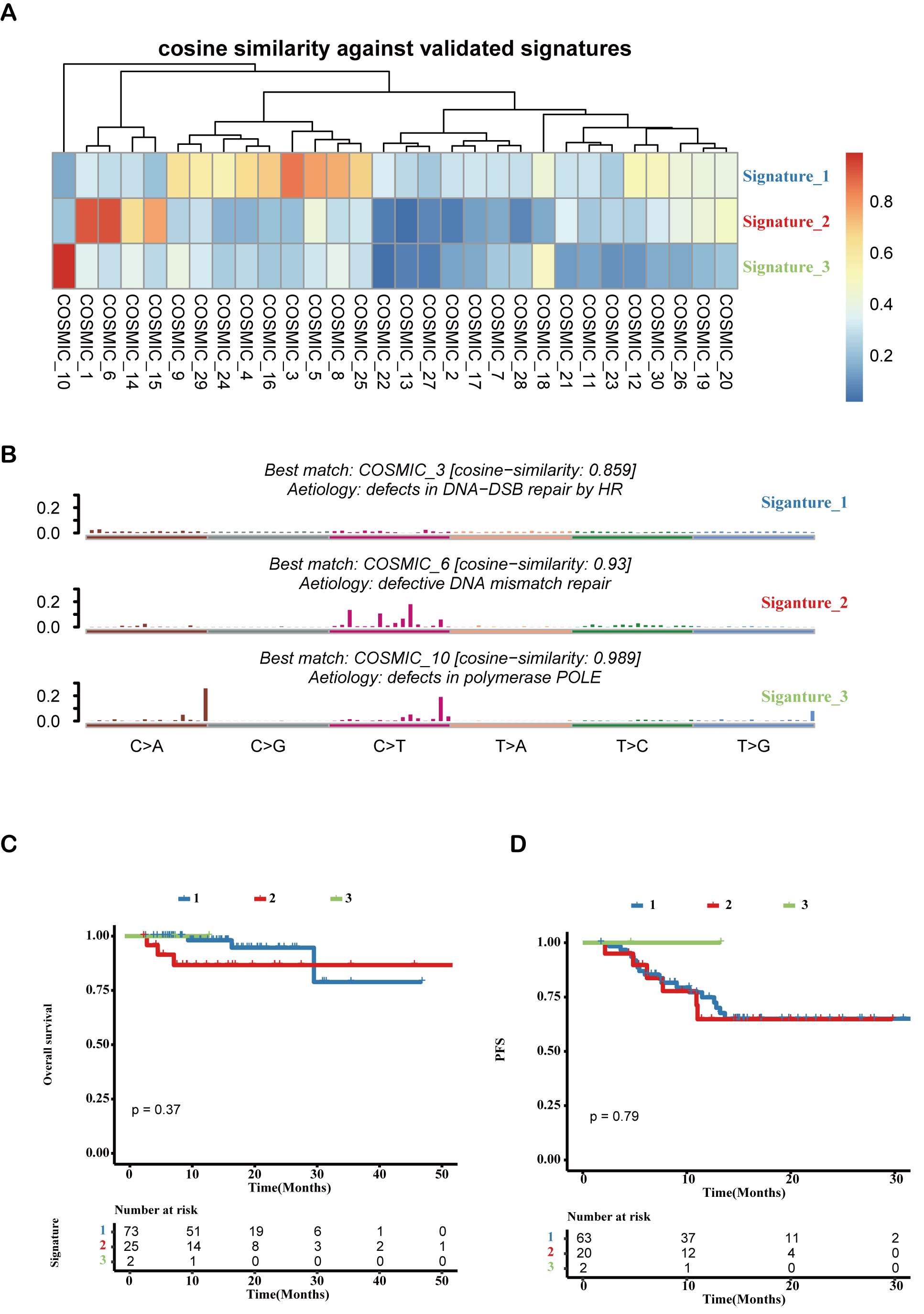


**Supplementary Figure 2**. The mutation signature of the FPHYP cohort. (**A**) Heatmap shows the cosine similarity between the three signatures and the COSMIC signatures. (**B**) The de novel mutational signatures exhibiting the closest resemblance to each COSMIC signature are denoted along with their cosine similarity score and biological relevance. (**C-D**) Kaplan-Meier curves were plotted to illustrate PFS (**C**) and OS (**D**) outcomes among the three distinct signature groups. COSMIC, Catalogue of Somatic Mutations in Cancer; PFS, progression-free survival; OS, overall survival.


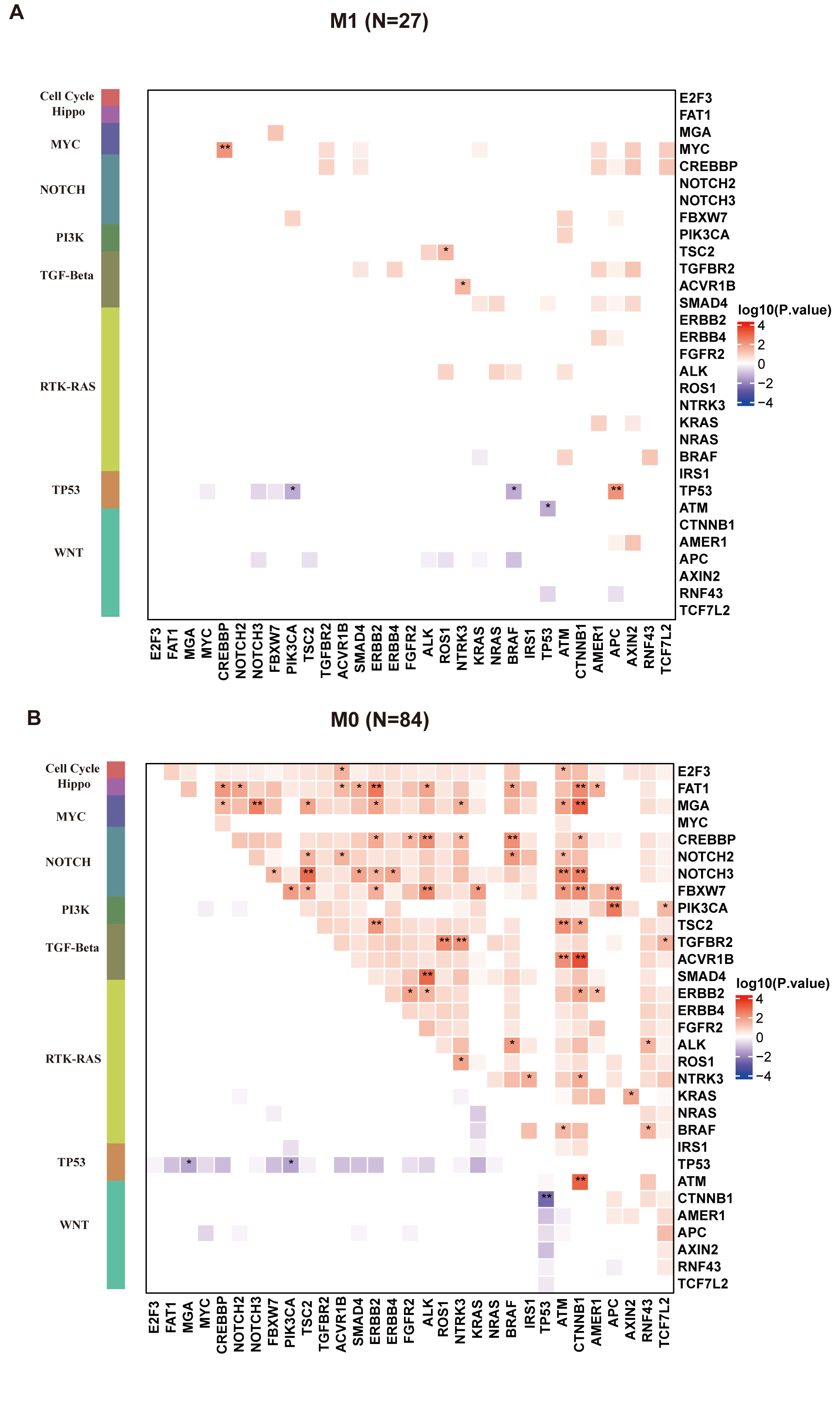


**Supplementary Figure 3**. **Co-mutational and mutual-exclusive features in non-mCRC and mCRC groups. (A-B)** The correlation plot illustrates the interplay between gene alterations within oncogenic signaling pathways, encompassing both co-occurrence and mutual exclusivity relationships of non-mCRC (**A**) and mCRC in the FPHYP cohort (**B**). * p < 0.05, ** p < 0.01. metastatic CRC; non-mCRC, non-metastatic CRC.

**
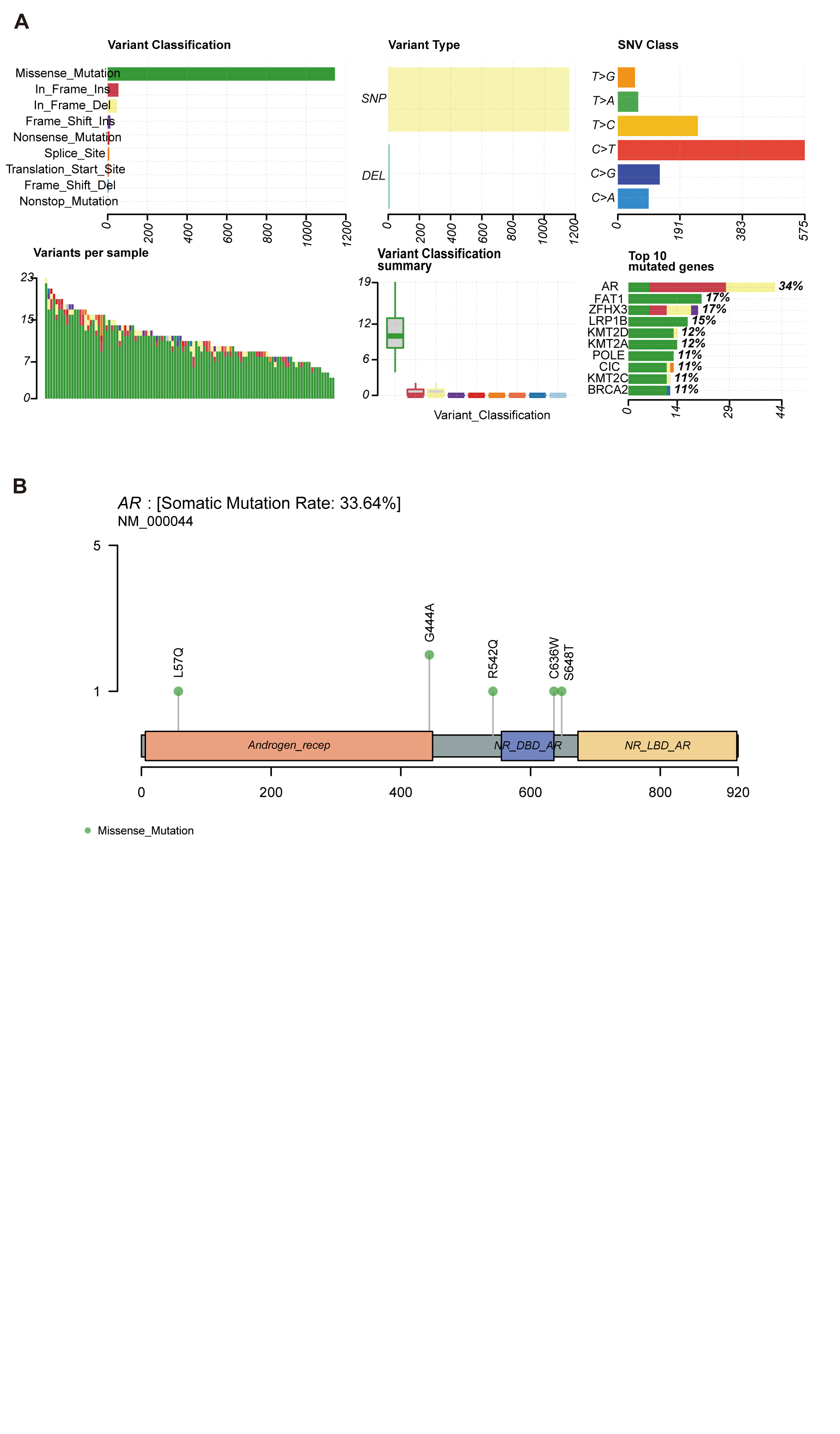
**

**Supplementary Figure 4. Germline alterations profile of CRC in FPHYP cohort.** (**A**) Classification of germline variant types is classified into distinct categories, with missense mutations constituting the predominant fraction. SNP exhibit a higher frequency compared to insertions or deletions (indels), with the most prevalent SNV being C>T. Variants are evaluated per sample. Additionally, the ten most frequently mutated genes in the germline are identified in CRC. (**B**)The lollipop plot of germline variants in *AR* gene. SNP, single nucleotide polymorphisms; SNV, single nucleotide variant.


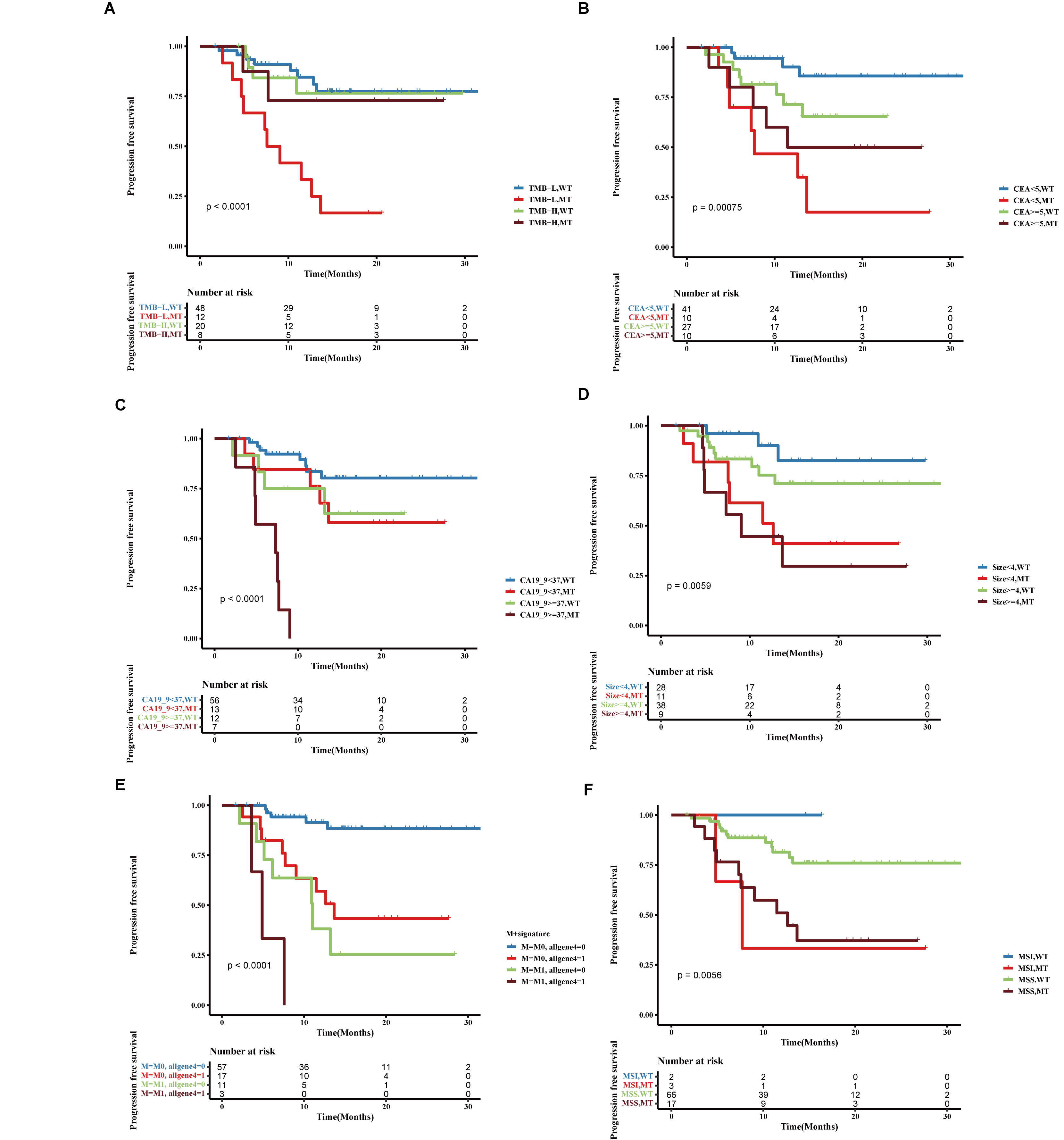


**Supplementary Figure 5**. **Stratified analysis was conducted by combining the mutation-based four-gene with clinical features. (A-F)** Kaplan-Meier survival curves of PFS survival among four groups stratified by the four-gene mutation signature and expression of TMB (**A**), CEA (**B**), CA19-9(**C**), tumor size(**D**), metastasis status (**E**), and the MSI status (**F**). PFS, progression-free survival; TMB, tumor mutation burden; CEA, carcinoembryonic antigen; CA19-9, carbohydrate antigen19-9; MSI, microsatellite instability.


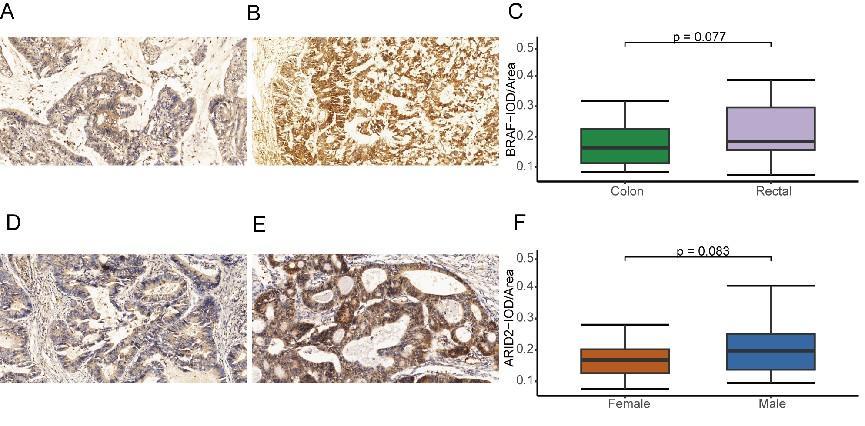


**Supplementary Figure 6**. **Immunohistochemical analysis of BRAF and ARID2 in CRC.** (**A-B**) The BRAF expression original field was acquired from tissue sections (magnification, 200x) of colon (**A**) and rectal tumor (**B**) location groups. (**C**) Comparison of the IOD/Area value between colon and rectal tumor location groups. (**D-E**) The ARID2 expression original field was acquired from tissue sections (magnification, 200x) of female (**D**) and male (**E**) groups. (**F**) Comparison of the IOD/Area value between female and male groups. IOD, cumulative optical density.
