# Supplementary material for: Comprehensive genomic profiling reveals prognostic signatures and insights into the molecular landscape of colorectal cancer
Source: Front Oncol. 2023 Nov 13;13:1285508. doi: 10.3389/fonc.2023.1285508 (PMC10680082; doi:10.3389/fonc.2023.1285508)
Supplement: Supplementary file 2 [file DataSheet_2.docx]

1. **Raw data**:

<https://www.jianguoyun.com/p/DZwub8oQwPXICxj5oZoFIAA>

1. **Publicly available data of MSKCC**: <https://www.jianguoyun.com/p/Df54n6kQwPXICxiAopoFIAA>
2. **R script**:

<https://www.jianguoyun.com/p/Deaf35sQwPXICxjKuoMFIAA>
